# Pediatric pan-central nervous system tumor analysis of immune-cell infiltration identifies correlates of antitumor immunity

**DOI:** 10.1038/s41467-020-18070-y

**Published:** 2020-08-28

**Authors:** Yura Grabovska, Alan Mackay, Patricia O’Hare, Stephen Crosier, Martina Finetti, Edward C. Schwalbe, Jessica C. Pickles, Amy R. Fairchild, Aimee Avery, Julia Cockle, Rebecca Hill, Janet Lindsey, Debbie Hicks, Mark Kristiansen, Jane Chalker, John Anderson, Darren Hargrave, Thomas S. Jacques, Karin Straathof, Simon Bailey, Chris Jones, Steven C. Clifford, Daniel Williamson

**Affiliations:** 1 Wolfson Childhood Cancer Research Centre, Northern Institute for Cancer Research, Newcastle University, Newcastle Upon Tyne, UK; 2 Division of Molecular Pathology and Division of Cancer Therapeutics, The Institute of Cancer Research, London, UK; 3 Department of Paediatric Oncology, Great Ormond Street Hospital NHS Trust, London, UK; 4 Developmental Biology and Cancer Programme, University College London Great Ormond Street Institute of Child Health, London, UK; 5 Department of Histopathology, Great Ormond Street Hospital for Children NHS Foundation Trust, London, UK; 6 UCL Genomics, UCL Great Ormond Street Institute of Child Health, London, UK; 7 Specialist Integrated Haematology and Malignancy Diagnostic Service-Acquired Genomics, Great Ormond Street Hospital for Children NHS Foundation Trust, London, UK

**Keywords:** Cancer genomics, Paediatric cancer

## Abstract

Immune-therapy is an attractive alternative therapeutic approach for targeting central nervous system (CNS) tumors and the constituency of the Tumor Immune Microenvironment (TIME) likely to predict patient response. Here, we describe the TIME of >6000 primarily pediatric CNS tumors using a deconvolution approach (methylCIBERSORT). We produce and validate a custom reference signature defining 11 non-cancer cell types to estimate relative proportions of infiltration in a panCNS tumor cohort spanning 80 subtypes. We group patients into three broad immune clusters associated with CNS tumor types/subtypes. In cohorts of medulloblastomas (*n* = 2325), malignant rhabdoid tumors (*n* = 229) and pediatric high-grade gliomas (*n* = 401), we show significant associations with molecular subgroups/subtypes, mutations, and prognosis. We further identify tumor-specific immune clusters with phenotypic characteristics relevant to immunotherapy response (i.e. Cytolytic score, *PDL1* expression). Our analysis provides an indication of the potential future therapeutic and prognostic possibilities of immuno-methylomic profiling in pediatric CNS tumor patients that may ultimately inform approach to immune-therapy.

## Abstract (web summary)

Here, using methylCIBERSORT, the authors characterize the tumour-immune microenvironment of paediatric central nervous system (CNS) tumours and its association with tumour type and prognosis. These findings suggest that immuno-methylomic profiling may inform immunotherapy approaches in paediatric patients with CNS tumour.

## Introduction

Immune therapies are an attractive alternative anti-cancer strategy alongside the conventional approaches of surgery, chemotherapy, and radiotherapy that may be particularly well suited to targeting diffuse infiltratively growing tumors. The field of cancer immunotherapy has grown expansively in recent years to include the therapeutic use of cancer vaccinations, chimeric antigen receptor T-cell therapy, and agents that block immune checkpoint receptors and/or ligand interactions such as CTLA-4 and PD-1. Each can provoke a significant antitumor response in patients within varied tumor types^1–7^. However, for each patient who derives clinical benefit from a particular immunotherapeutic agent, there are many whom do not^8^. The composition of the tumor immune microenvironment (TIME) is a critical determinant of tumor–immune interactions and can direct response to treatment^9^. Therefore, to take full advantage of the potential of immunotherapy—or combinations with targeted agents—treatment approaches need to be tailored to the specific TIME.

Detailed studies of the TIME are being conducted to predict response to immunotherapy and uncover mechanisms of treatment resistance. Although anti-PD-1 antibodies nivolumab and pembrolizumab, and an anti-CTLA-4 antibody Ipilimumab are Food and Drug Administration approved and can produce durable responses in patients with metastatic melanoma^10–12^, non-small cell lung cancer^13^, and renal cell carcinoma^14^, the majority of patients do not respond. Comparative studies between responders and non-responders indicate that multiple factors, including pre-existing T-cell infiltration, checkpoint molecule expression within the tumor, and mutational burden with consequent production of neoantigens correlate with response to immune therapy. For instance, colorectal cancer of the molecular subtype CMS1 are characterized by DNA mismatch-repair defects, microsatellite instability, and hypermutation with accompanying infiltration of CD8+ T-cells^15^ and expression of immune checkpoint proteins CTLA-4, PD-1, PDL1, and IDO-1^16–18^. CMS1 patients show significant responses to anti-PD-1 therapies^19^.

Tumors are frequently described as being immunologically “hot” or “cold” with a presumed implication for the effectiveness of particular tumor immune therapies. “Hot” tumor TIMEs are broadly characterized by high expression of the PD-1 ligand (PDL1) and by infiltration of cytotoxic lymphocytes (CTLs) expressing PD-1. “Cold” tumors being relatively sparsely infiltrated with CTLs, at least within the tumor core^8^. Childhood brain tumors are thought to be relatively immunologically “cold” due to paucity of mutations (i.e., generally lacking neoantigens^20^). To date, quite limited information on TIME in childhood brain tumors has been published and in piecemeal manner. In adult brain tumors, several immune cell types have identified roles in, and associations with, tumor development. For instance, tumor-associated macrophages are believed to make up a large proportion of immune cells in gliomas^21^ and to be generally pro-tumorigenic and associated with a higher tumor grade^22,23^. Furthermore, the number of neutrophils appears to have prognostic value^24,25^ and immuno-suppressive regulatory T-cells (Treg) are significantly increased in patients with glioma as a proportion of the peripheral CD4+ cell pool; they also account for a substantial proportion of the TIME^26,27^. Simple extrapolation from adult brain tumors is unlikely to be informative given the underlying differences in tumor biology.

A number of methods exist to characterize and quantify TIME directly, e.g., immunohistochemistry (IHC), fluorescence-assisted cytometry (FACS), cytometry by time-of-flight (Cy-TOF), and single-cell RNA sequencing (RNA-seq). These may be costly, laborious, and/or difficult to multiplex. Indirect techniques have been developed to estimate TIME in silico by deconvoluting complex mixtures of cell types from profiles of bulk populations using pure populations of cell types as a reference^28–30^. CIBERSORT is a notable algorithm that uses support vector regression modeling to deconvolute cell types and has been applied to several cancer datasets^28^. Central nervous system (CNS) tumors have been extensively DNA methylation profiled using arrays, most prominently by Capper et al.^31^ who published a cohort of 3764 CNS tumors (including 1403 patients < 18 years old) representing 80 tumor DNA methylation types and subtypes closely related to World Health Organization (WHO) histopathological entities. We and others have published further large series of some of the major pediatric CNS types, i.e., medulloblastoma (MB)^32–35^, atypical teratoid/rhabdoid tumors (ATRT)^36,37^, and pediatric high-grade gliomas (pHGG)^38,39^ with extensive clinical annotation and parallel multiomic data (RNA-seq, copy-number profiles, exome/whole-genome sequencing). Here we use methylCIBERSORT—a recent adaptation of the CIBERSORT algorithm, which uses genome-wide DNA methylation data^40^—to characterize the TIME of >6000 CNS tumors, assessing variation and the relationship with clinico-pathology or outcome. 850K methylation arrays are currently employed in several countries as part of a standard diagnostic workup. We show that the ability to characterize multiple cell types in a single experiment and generate immune infiltrate estimates from the same data generates significant added value.

## Results

### Generation of a signature matrix for cellular deconvolution

**Fig. 1:**
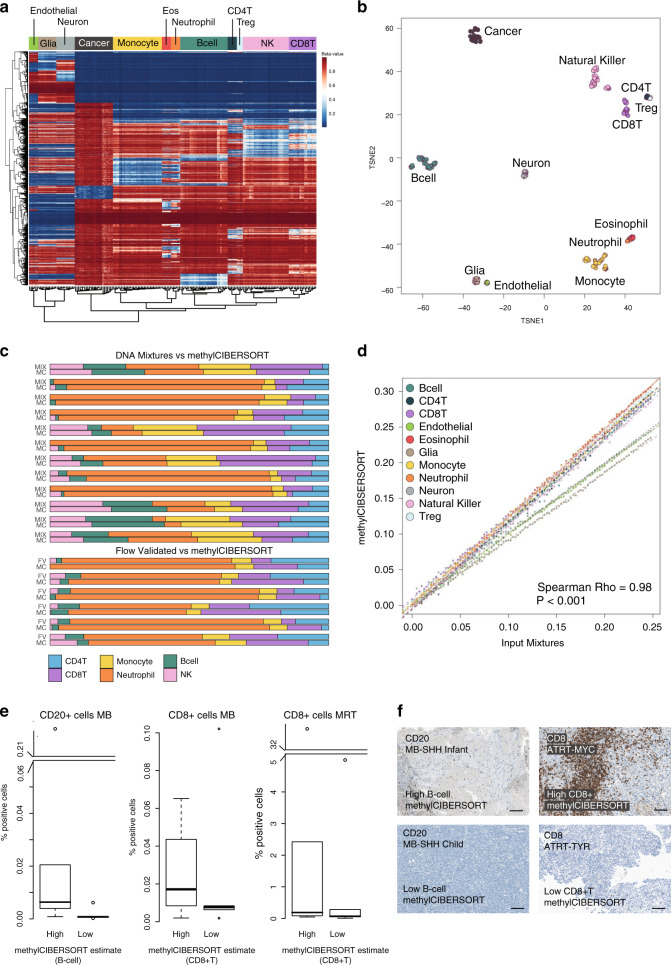
Generating and benchmarking the signature matrix. **a** Heatmap of *β*-values for CpGs (rows) and samples (columns) used in the methylCIBERSORT signature matrix. Columns/rows are ordered by unsupervised hierarchical clustering which independently resolves reference cell type. **b** t-SNE plot showing methylation profiles of the pure reference cell types. **c** Barplot showing the methylCIBERSORT estimates of immune-cell-type proportions (MC) vs. known flow-validated proportions in six control PBMC cell mixtures (FV) or artificial mixtures generated from combined known proportions of isolated immune cell types (MIX). **d** Scatterplot showing a significant correlation (Pearson, *ρ* = 0.98, *p* < 0.001, *n* = 1100) between known input and methylCIBERSORT estimates of randomly simulated cell mixtures created in silico to contain 75% cancer and known proportions of input cell types. **e** Boxplots showing results of immunohistochemical (IHC) staining of medulloblastoma (MB) and malignant rhabdoid tumor (MRT) tissue sections with antibodies for CD20 and CD8; *n* = 30 biologically independent samples. % positive cells are shown by “High” and “Low” categories. “High” represents five samples (for which tissue sections were available) with the highest methylCIBERSORT estimate for either B-cells or CD8+ T-cell infiltration samples. “Low” represents five samples for which a methylCIBERSORT estimation of B-cells or CD8+ T-cell infiltration was 0 or negligible. Discontinuous axes are used where needed to represent outliers. Data represents the % positive cells from a minimum of 15,000 cells assessed per sample (median cells examined = 826,375). Box represents interquartile range, center line represents median, whiskers represent range of minima and maxima excluding outliers, which are represented as points. **f** Images of IHC staining showing examples from the aforementioned “High” and “Low” categories. Scale bar represents 100 μM.

We first constructed a signature matrix from reference DNA methylation profiles of pure flow-sorted populations of cells. This signature matrix represents a set of differentially methylated CpGs selected and weighted to reflect specificity for a given cell type and is used as the basis of cell deconvolution by methylCIBERSORT. Our final signature matrix consisted of 2215 differentially methylated CpGs distinguishing between 12 broad cell types: Tregs, CD4+ T-cells (CD4T), CD8+ T-cells (CD8T), B-cells (B-cell), natural killer (NK) cells, eosinophils, neutrophils, monocytes, endothelial cells, glial cells, neurons, and cancer. Where cancer represents relevant cancer cell line profiles from multiple tumor types (see Supplementary Table 1), we verified the following: (i) that specific differentially methylated CpGs were captured for each cell type, (ii) the absence of batch effects following processing; and (iii) the CpGs selected were not confounded by being specific to any particular CNS cancer type (Fig. 1a, b and Supplementary Fig. 1).

We benchmarked our new signature matrix using publicly available methylation profiles of peripheral blood mononuclear cells (PBMCs) with known cell composition as determined by flow cytometry or constituted from mixtures of reference DNAs of known proportions. We found a significant level of correlation between our methylCIBERSORT estimates and the flow cytometry measurements and known DNA mixtures (*ρ* = 0.84, *p* < 0.001, *n* = 36 and *ρ* = 0.91, *p* < 0.001, *n* = 72, respectively, Fig. 1c). We also tested 100 synthetic mixtures for each cell type generated in silico using methylation profiles of random pure cell populations mixed 1 : 4 with a mixture of cancer cell line profiles (Fig. 1d). Again, there was a highly significant correlation between estimated and actual cell composition (*ρ* = 0.98, *p* < 0.001, *n* = 1100). Finally, we measured the extent of immune cell tumor infiltration using IHC (CD20, CD8) in a subset of our tumor samples (*n* = 30). We were able to validate our methylCIBERSORT estimates of B-cell and CD8+ T-cells against our IHC-based estimates (Fig. 1e, f).

### PanCNS tumors show significant differences in TIME by type

We next applied methylCIBERSORT to a set of 3764 panCNS tumor methylation profiles (plus an additional 141 control/hematopoietic samples, see Supplementary Table 4) published by Capper et al.^31^. This reference set is the training resource of the Molecular Neuro-Pathology 2.0 classifier and represents 80 methylation tumor types/subtypes closely related to WHO histopathological entities and divided into 13 broad histological categories. A relative proportion of the 12 cell types were estimated and indicators of deconvolution performance examined. As further validation, the relative proportion of cancer cells estimated by methylCIBERSORT was significantly correlated with the estimate of tumor purity provided by Capper et al.^31^ (based on machine learning estimates trained on a set of known glioma positives) (*ρ* = 0.71, *p* < 0.01, *n* = 3784, Supplementary Fig. 4A). The sum of the estimated proportions of all tumor-infiltrating lymphocytes (TILs) (i.e., Treg, CD4T, CD8T, and NK) correlates significantly with the meTIL score (an independent measure of T-lymphocyte infiltration based upon methylation status of 5 CpGs) defined by Jeschke et al.^41^ (*ρ* = 0.29, *p* < 0.001, *n* = 3764, Supplementary Fig. 4B). As expected, control samples having a known inflammatory or reactive tumor microenvironment were associated with a large increase in the estimated median proportion of neutrophils (86% vs. 0%, *W* = 0, *p* < 0.001) and monocytes (50% vs. 17%, *W* = 17, *p* < 0.001), respectively, compared to the average of other CNS control tissues (Supplementary Fig. 4C).

Calculating the median estimated relative proportions of non-cancer cell types showed that on average across all CNS tumor types the largest fractions of non-cancer cells proportionally were Tregs (20% of all non-cancer cells) and monocytes (20%) followed by B-cells (16%), CD8T (14%), eosinophils (12%), NK cells (12%), CD4T (9%), and neutrophils (8%). Relatively modest proportions of neuronal (3%), endothelial cells (2%), and glia (1%) were estimated.

**Fig. 2:**
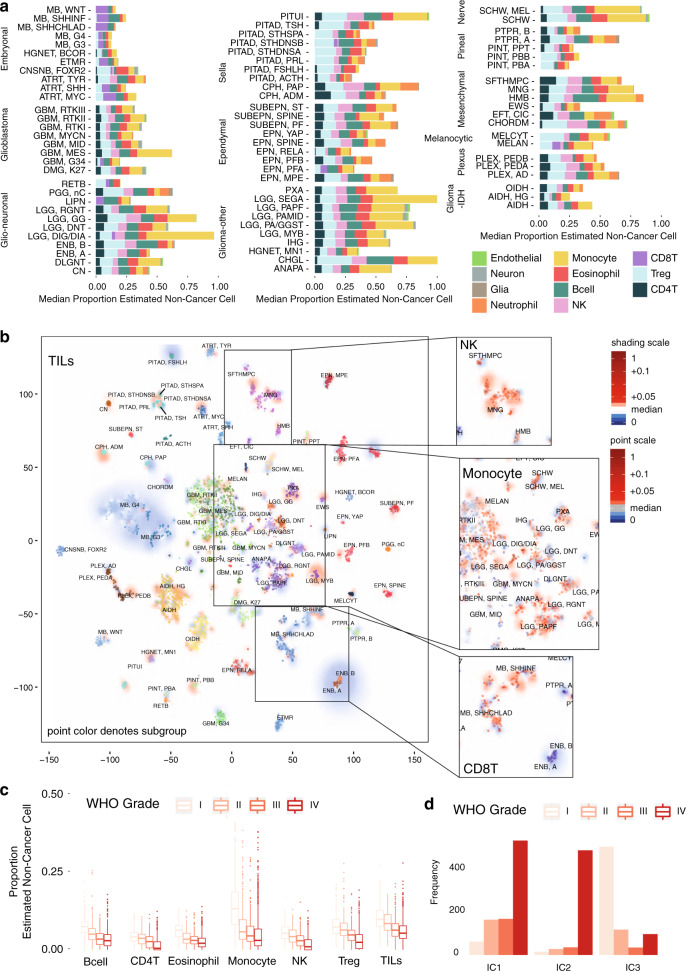
Deconvolution analysis of panCNS tumor immune infiltration. **a** Barplots of the estimated median infiltration of specific cell types as a proportion of all non-cancer cell types (range scaled from 0 to 1) in 3763 CNS tumor samples from the Capper et al.^31^ panCNS tumor cohort. Data shown by tumor type/subtype highlighting the range and variation of immune cell infiltration in different CNS tumor types. **b** t-SNE plot representing the methylation profiles of the panCNS cohort. The colors of dots and a text label marking tumor type/subtype in the central panel map to the tumor type are as per Capper et al.^31^ (for full key see Supplementary Table 4). Background shading represents the 2D spatial density estimation of the amount of tumor-infiltrating lymphocytes (TILs); red shading equals relatively greater than average infiltration and blue shading equals less than average. Exploded side panels represent enlarged areas of interest wherein both dot color and background shading represent the relative amount of the particular immune cell infiltration denoted. Red color denotes relatively greater than average infiltration and blue color denotes less than average. **c** Boxplot showing a negative association between proportion of estimated cell types and WHO grade; *n* = 2315 biologically independent samples. Box represents interquartile range, center line represents median, and whiskers represent range of minima and maxima excluding outliers that are represented as points. **d** Barchart showing differences in frequency of patients of different WHO grade by immune cluster; *n* = 2315 biologically independent samples.

Individual tumor types/subtypes varied significantly in the relative proportions of infiltrating cell types; each cell type was significantly non-randomly distributed with respect to tumor type/subtype (as calculated by Kruskal–Wallis (KW) one-way analysis of variance, each *p* < 0.001, see Supplementary Data 1) (Fig. 2a, b). Post hoc testing (Dunn’s test) reveals the relative number of TILs and indeed the total amount of infiltrating cells was significantly less in high-grade tumor types such as embryonal tumors (i.e., MB, ATRT, and Embryonal tumours with multilayered rosettes) than in low-grade gliomas (LGGs) (*p* < 0.001). Examining the median relative proportions of the 11 non-cancer cell types across CNS tumors, those with the greatest variance are monocytes, Tregs, and CD8T. Notably, LGG subtypes have a proportionally greater number of monocytes, making up an estimated 35% of all infiltrating cells (Supplementary Fig. 4D) compared to 13% in embryonal tumors. CD8T, e.g., is proportionally greater in MB_Grp3_ and MB_SHHCHLD_, making up an estimated 48% and 40% of all infiltrating cells, respectively, compared to 6% in LGG. Tregs are relatively greater proportionally in the Sellar tumors (specifically pituitary adenomas) constituting an estimated 36% of all infiltrating cells compared to 14% in glioblastoma and 17% in embryonal tumors (Fig. 2b and Supplementary Fig. 4D).

Consensus clustering of immune cell estimates identifies an optimal three immune clusters we refer to as panCNS_IC1-3_. Members of panCNS_IC1_ have a relatively high proportion of Tregs and a relative lack of CD8T cells. panCNS_IC2_ have a relatively high proportion of CD8T and low proportions of CD4T/Tregs and NK cells. panCNS_IC3_ has a relatively high proportion of monocytes and relative lack of CD8T (Supplementary Fig. 4E). Membership of an immune cluster was related to but by no means exclusively dictated by tumor type. Although immune cluster is significantly non-random with respect to tumor subgroup/subtype (*χ*^2^ =3303, *p* < 0.001), most tumor subgroups cut across multiple immune clusters to some extent (Supplementary Fig. 5A).

The significance of association with available clinico-pathological characteristics (WHO grade, disease stage, i.e., metastases/relapse/diagnosis, age category, i.e., <3 years/3–16 years/>16 years, tumor location, gender) was assessed and the proportion of immune cell types was significantly associated with each of the clinico-pathological characteristics with the exception of gender (see Supplementary Data 1). The strongest association was with WHO grade for which the average infiltration of certain immune cell types (eosinophils, CD4T, B-cell, Treg, NK, monocytes, and TILs) decreases proportionally with increasing WHO Grade (I–IV) (Fig. 2c and Supplementary Fig. 4E). Immune cluster membership is significantly associated with WHO grade (*χ*^2^ = 1249.3, *p* < 0.01). Eighty-seven percent (509/587) of all WHO Grade I tumors belong to panCNS_IC3_ and panCNS_IC2_ consists of 86% (492/571) Grade IV tumors (Fig. 2d). Such associations are unsurprising given the strong interdependence of clinico-pathological factors with tumor subtype. However, a regression analysis using only tumor types for which grade, age category, and tumor location were variable showed a number of clinico-pathological associations significant independently of tumor subgroup (see Supplementary Data 1). B-cells, CD4T, eosinophils, and Tregs were each significantly negatively associated with tumor stage (each *p* < 0.01) independently of the subgroup. Monocytes were also significantly positively associated with spinal location independent of the subgroup. In summary, this analysis reveals the existence of at least three distinct TIME classes across CNS tumors strongly related to but not exclusively dictated by tumor subgroup and grade.

### Medulloblastoma TIME is related to molecular subtype

**Fig. 3:**
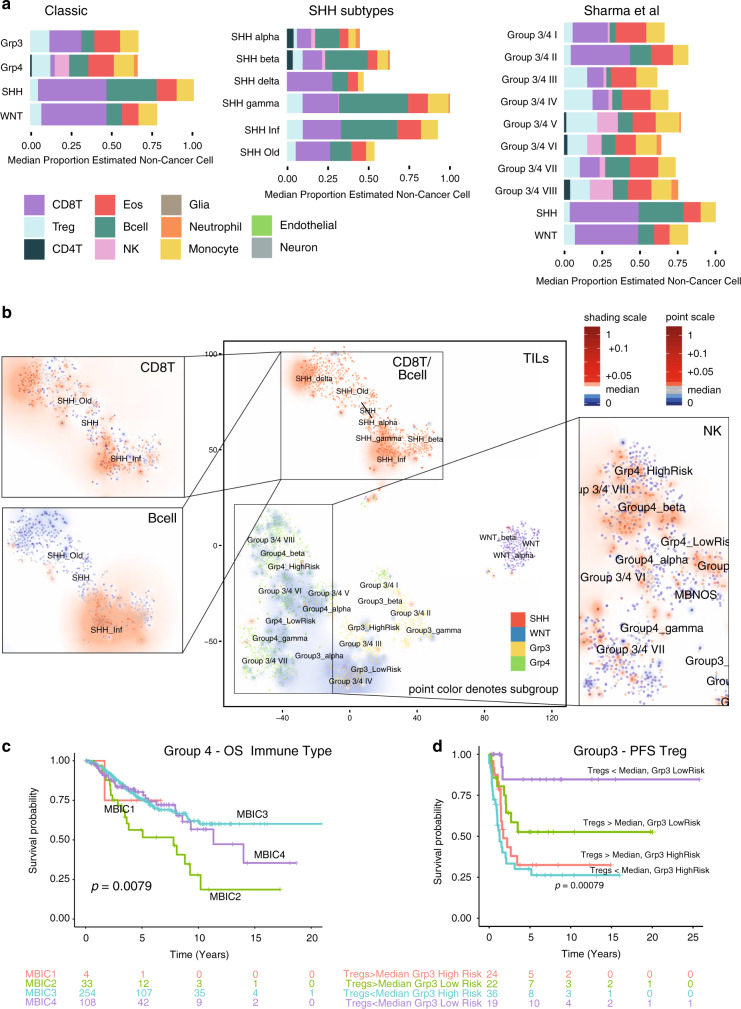
Deconvolution analysis of medulloblastoma immune infiltration by subgroup. **a** Barplots of the estimated median infiltration of specific cell types as a proportion of all non-cancer cell types (range scaled from 0 to 1) in 2325 medulloblastoma by subgroup (classic 4 medulloblastoma consensus subgroups^42^) by SHH subtype^32,33^ and by 10 group consensus as per Sharma et al.^35^. **b** t-SNE plot representing the methylation profiles of 2325 medulloblastoma. The colors of dots in the central panel map to the classic four molecular subgroups: red, SHH; blue, WNT; yellow, Grp3; green, Grp4. Text represents centroids of individual subtypes as reported variously by Cavalli et al.^33^, Sharma et al.^35^, and Schwalbe et al.^32^. Background shading represents the 2D spatial density estimation of the amount of tumor-infiltrating lymphocytes (TILs); red shading equals relatively greater than average infiltration and blue less than average. Exploded side panels represent enlarged areas of interest wherein both dot color and background shading represent the relative amount of the particular immune cell infiltration denoted. Red denotes relatively greater than average infiltration and blue less than average. **c** Kaplan–Meier plot showing significant difference in overall survival in MB_Grp4_ by immune cluster; log-rank *p* = 0.008, *n* = 399. **d** Kaplan–Meier plot showing significantly different progression-free survival (PFS) within the MB_Grp3_ Schwalbe et al.^32^ subtypes by low (<median) or high (>median) levels of Treg infiltration; log-rank *p* < 0.001, *n* = 101.

We next sought to characterize a more refined TIME in a single tumor type. By applying methylCIBERSORT to a set of 2325 MB methylation profiles, published by ourselves and others^32–35^, for which more detailed clinico-pathological and parallel multiomics data were available. Each of these studies elaborated upon the four classic subgroups of MB (MB_WNT_, MB_SHH_, MB_Grp3_, and MB_Grp4_)^42^ to describe further derivative subtypes including high-risk or low-risk subtypes of MB_Grp3/Grp4_. The most abundantly estimated infiltrated non-cancer cell types on average across all MB subgroups were CD8T (27% of all non-cancer cells), B-cells (16%), and eosinophils (15%). The proportion of each cell type was significantly different with respect to the four classic subgroups (all *p* < 0.001, see Supplementary Data 1) and post hoc testing shows significantly greater CD8T in MB_Grp3_ vs. MB_Grp4_ (7.3-fold, *p* < 0.001), greater NK in MB_Grp4_ vs. other subgroups (9.7-fold, all comparisons *p* < 0.001), and greater B-cells in MB_SHH_ vs. other subgroups (3-fold, all comparisons *p* < 0.001) (Fig. 3a, b).

We recently published a meta-analysis describing a further refinement of the MB_Grp3/Grp4_ subgroups into eight subtypes I–VIII^35^. These subtypes are also associated with differences in estimated levels of each cell type with the exception of monocytes (each *p* < 0.002, see Supplementary Data 1). Post hoc analysis shows the most significant differences to be CD8T (greater in subtype II), Tregs (less in subtype II), NK (greater in subtype VIII), and B-cells (less in subtype III) (all comparisons *p* < 0.01) (Fig. 3a, b). Significant differences were apparent between MB_SHH_ subtypes. Both the infant SHH subtype (described by Schwalbe et al.^32^) and the SHH γ-subtype (described by Cavalli et al.^33^) show significantly greater proportions of B-cells than other MB_SHH_ subtypes (2.6- and 2.5-fold, respectively, both *p* < 0.001) (Fig. 3a, b).

Consensus clustering of MB immune cell estimates identifies an optimal four immune clusters referred to here as MB_IC1-4_, which cut across each of the MB subgroups/subtypes (Supplementary Fig. 6A). These amount to further refinements of the immune clusters defined in the panCNS tumor analysis with MB_IC1_/MB_IC4_ overlapping primarily with panCNS_IC2_ and MB_IC2_/MB_IC3_ overlapping primarily with panCNS_IC1_ (Supplementary Fig. 5B)_._ MB_IC1_ constitutes 7% (167/2325) of all MB and is characterized by relatively high proportions of B-cells and CD8T, and a disproportionately high number of MB_SHH_ patients; 83% of MB_IC1_ are also MB_SHH_ (*χ*^2^ = 425.59, *p* < 0.001). MB_IC2_ constitutes 7% (162/2325) of all MB and is characterized by relatively high proportions of Treg, eosinophils, and NK, and low proportions of CD8T. MB_IC3_ constitutes 42% (981/2325) of all MB, has relatively low proportions of CD8T, relatively moderate levels of all other infiltrating immune types, and a disproportionately high proportion of MB_Grp3/Grp4_ (78% of MB_IC3_). MB_IC4_ constitutes 44% (1015/2325) of all MB and is characterized by a relatively high proportion of CD8T cells and relatively low–moderate levels of other infiltrating immune cell types (Supplementary Fig. 6A).

Further associations between infiltrating cell estimations and clinico-pathological variables (within the four classic subgroups) were examined includingthe following: *MYC*/*MYCN* amplification, *TP53* mutation, and metastatic stage (see Supplementary Data 1). *MYC* amplification in MB_Grp3_ was associated with a significantly higher proportion of TILs, CD8T, and B-cells (KW = 8.7, 16.7, 18.9, respectively, each *p* < 0.01, *n* = 408), and a lower infiltration of Tregs (KW = 11, *p* = 0.012, *n* = 408) (Supplementary Fig. 6B).

Estimated immune cell infiltration was examined for association with survival in each subtype (excluding MB_WNT_). Membership of MB_IC2_ was associated with poorer overall survival (OS) in MB_Grp4_ (log-rank *p* = 0.0079, *n* = 399) (Fig. 3c). Cox regression shows several individual cell types are significantly associated with outcome (see Supplementary Data 1). In some instances, immune cell estimates provide prognostic information independent of previously established survival associated methylation subtypes^32^. For instance, a greater than median proportion of monocytes in MB_Grp4_ is associated with a poor prognosis (OS: hazard ratio (HR) = 1.7, CI95%^upper^ = 2.5, CI95%^lower^ = 1.2, *p* = 0.006, *n* = 399; progression-free survival (PFS): HR = 1.9, CI95%^upper^ = 3.7, CI95%^lower^ = 1.0, *p* = 0.039, *n* = 133). Multivariate analysis shows that this association is significantly prognostic, independent of the MB_Grp4_ high-risk/low-risk subgrouping of Schwalbe et al.^32^ (OS: HR = 2.2, CI95%^upper^ = 4.5, CI95%^lower^ = 1.1, *p* = 0.023, *n* = 135; PFS: HR = 2.0, CI95%^upper^ = 3.8, CI95%^lower^ = 1.1, *p* = 0.032, *n* = 133) (Supplementary Fig. 6C). For infant MB_SHH_, a greater than median proportion of Tregs was significantly associated with a poor PFS (OS: HR = 3.3, CI95%^upper^ = 8.9, CI95%^lower^ = 1.2, *p* = 0.021, *n* = 64; PFS: HR = 2.7, CI95%^upper^ = 6.4, CI95%^lower^ = 1.1, *p* = 0.029, *n* = 59) (Supplementary Fig. 6D). Likewise, the proportion of Tregs distinguishes two groups within the previously described MB_Grp3_ low-risk subtype^32^ with significantly different survival (log-rank *p* < 0.001, 5yrEFS 88% vs. 52%) (Fig. 3d). This demonstrates that immune infiltration estimates are able to add additional prognostic information not readily available from previous methylation-based analysis.

In order to independently validate our findings, we analyzed, a subset of MB samples for which we possessed both methylation and RNA-seq data. We calculated the expression-based metric “Cytolytic score” (CYT = the mean expression of *GZMA* and *PRF1*) as described by Rooney et al.^43^ and showed that this was significantly correlated with methylCIBERSORT estimates of TILs (*ρ* = 0.18, *p* = 0.015, *n* = 185) and differed significantly by immune cluster (*F* = 4.1, *p* = 0.008, *n* = 185) being greatest in MB_IC1_ and poorest in MB_IC3_ (Supplementary Fig. 6E). Expression of immune checkpoint genes *PDL1* and *CD276* were also significantly different with respect to immune clusters (both *p* < 0.01); MB_IC1_ in particular showed high expression of *PDL1* and low expression of *CD276*. We used ssGSEA analysis and gene sets, which define two of the largest and most variable infiltrating cell types B-cell and CD8T, to create a per-patient metagene score, which summarizes the strength of the relevant expression signature. This was performed in both our RNA-seq cohort and a matched Affymetrix expression array cohort (*n* = 763) each was significantly correlated with their equivalent methylCIBERSORT estimates (each *p* < 0.01). Matching enrichment in CD8T expression signatures were found in MB_SHH_, MB_WNT_, and MB_Grp3/4_ subtypes and increases in B-cell expression signatures within MB_SHH_ and MB_Group3/4 II_ supports the methylCIBERSORT estimates (Supplementary Fig. 7A, B). Likewise, the relative lack of CD8T in MB_IC3_ and B-cell enrichment in MB_IC1_ is mirrored by the expression signature analysis (Supplementary Fig. 7C, D).

### MRT TIME is associated with subtype, location, and prognosis

**Fig. 4:**
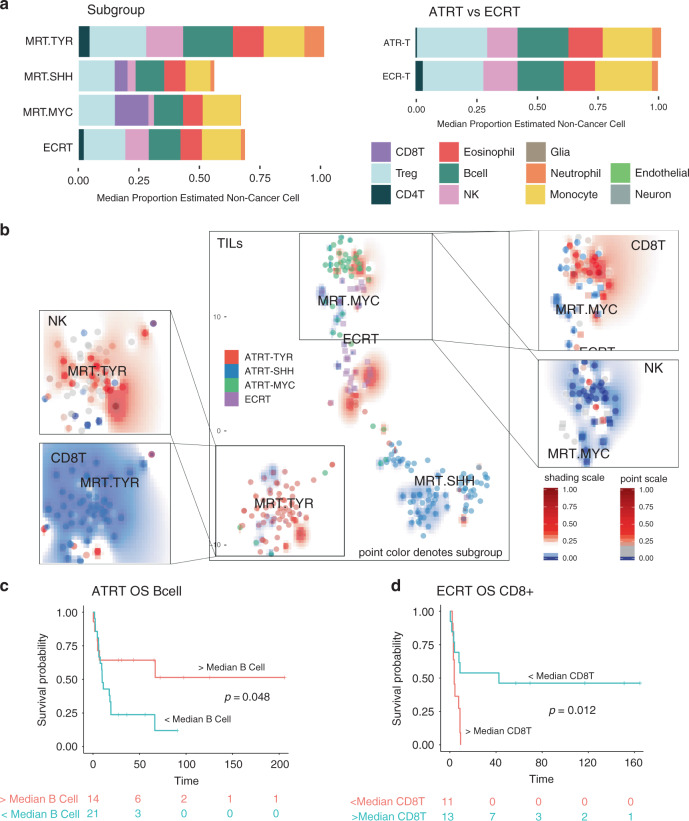
Deconvolution analysis of malignant rhabdoid tumors immune infiltration. **a** Barplots of the estimated median infiltration of specific cell types as a proportion of all non-cancer cell types (range scaled from 0 to 1) in 229 malignant rhabdoid tumors (MRTs) by subgroup (three molecular subgroups originally described by Johann et al.^36^) and by tumor location. ATRT, atypical teratoid/rhabdoid tumor (within the CNS, i.e., intracranial); ECRT, extra-cranial rhabdoid tumor. **b** t-SNE plot representing the methylation profiles of 229 MRT. The colors of dots in the central panel map to molecular subgroups (as per Johann et al.^36^). Text represents centroids of individual subtypes. Background shading represents the 2D spatial density estimation of the amount of tumor-infiltrating lymphocytes (TILs); red shading equals relatively greater than average infiltration and blue less than average. Exploded side panels represent enlarged areas of interest wherein both dot color and background shading represent the relative amount of the particular immune cell infiltration denoted. Red denotes relatively greater than average infiltration and blue less than average. **c** Kaplan–Meier plot showing significantly different overall survival (OS) in ATRT with > or <median numbers of B-cells; log-rank, *p* = 0.048, *n* = 35. **d** Kaplan–Meier plot showing significantly different overall survival (OS) in ECRT with > or <median numbers of CD8+ T-cells; log-rank, *p* = 0.012, *n* = 24.

We next ran our methylCIBERSORT on a set of 229 malignant rhabdoid tumor (MRT) methylation profiles from a previously published study^36^ and supplemented with 79 previously unpublished profiles. The MRT cohort was made up of 192 ATRT samples and 37 extra-cranial rhabdoid tumors (ECRT). MRT are on average infiltrated predominantly by Tregs (19% of non-cancer cells), monocytes (18%), B-cells (15%), and CD8T (13%) (Fig. 4a, b). Taking the three previously described molecular subgroups of ATRT (ATRT-TYR, ATRT-SHH, and ATRT-MYC^36^) and ECRT, the distribution of each estimated immune cell type is significantly different with respect to ATRT subgroup (all *p* < 0.05) (Fig. 4a, b and Supplementary Fig. 8B). Post hoc testing shows the most significant are NK, Treg, B-cells (each greater in ATRT-TYR), and CD8T (significantly greater in ATRT-MYC and ATRT-SHH) (Fig. 4a, b and Supplementary Fig. 8B). Surprisingly, no immune cell types were found to be significantly different overall between ATRT (all subtypes) and ECRT (Fig. 4a).

Consensus clustering of MRT immune cell infiltration estimates identifies four robust immune subgroups, which cut across the tumor subgroups and are named MRT_IC1-4._ These amount to further refinements of the immune clusters defined in the panCNS tumor analysis with MRT_IC2_ overlapping primarily with panCNS_IC1_ and MRT_IC4_ overlapping primarily with panCNS_IC2_ (Supplementary Fig. 5C). MRT_IC1_ and MRT_IC3_ constitute minor clusters, only 2% (4/229) and 6% (14/229) of all MRT, and have relatively high proportion of neutrophils and monocytes, respectively. Both clusters contain a disproportionate number of ECRT and ATRT-TYR (*χ*^2^ = 48.218, *p* < 0.001) (Supplementary Fig. 8A). MRT_IC4_ constitutes 32% (74/229) of all MRT and is characterized by a relatively high proportion of CD8T and relatively low infiltration of other immune cell types. MRT_IC2_ constitutes 60% (137/229) of all MRT and is characterized by a relative lack of CD8T and relatively moderate infiltration of other immune cell types; 83% (59/71) of ATRT-TYR are of this type.

Examining the association with outcome in ATRT, we found a greater than median level of B-cells was associated with a significantly improved OS (log-rank, *p* = 0.048, *n* = 35) (Fig. 4c). In ECRT, a greater than median level of CD8T was associated with a significantly poorer OS (log-rank *p* = 0.012, *n* = 24) (Fig. 4d). It should be noted that molecular subgroup alone was non-significant with respect to OS in both ATRT and ECRT.

No significant differences in immune infiltration are seen with respect to age category (<2 vs. >2 years), the presence of metastases at diagnosis, and type of *SMARCB1* mutation (Supplementary Data 1). The only significant clinico-pathological association is a lower proportion of monocytes and a higher proportion of NK cells in infratentorial compared to supratentorial ATRT (*W* = 1469.5 and *W* = 2726.5, respectively, both *p* < 0.001) (Supplementary Fig. 8C, D).

To validate our methylCIBERSORT results, a CYT score was again calculated in samples for which parallel RNA-seq data were available. This was significantly correlated with methylCIBERSORT estimates of TILs in MRT (*p* < 0.01, *n* = 28) (Supplementary Fig. 9A). We also used mixcr^44^ analysis to identify non-germline (i.e., definitively rearranged and therefore originating from T-cells or B-cells) BCR and TCR reads within the RNA-seq data and thus CDR3 clonotypes. The total normalized TCR and BCR read counts correlated significantly with the methylCIBERSORT estimates of T-lymphocytes and B-cells, respectively (each *p* < 0.05, Supplementary Fig. 9B, C). MRT_IC2_ had a 1.8-fold lower mean CYT score and 1.7-fold greater mean *PDL1* expression than that of MRT_IC4_ (Supplementary Fig. 9D). Using the same ssGSEA approach as for MB shows enrichment of CD8T and B-cell expression signatures matching methylCIBERSORT estimates according to subtype (Supplementary Fig. 9E, F). Taken as a whole, this underlines the significant relationships between subgroup, prognosis, and immune cell infiltration in MRT.

### TIME is associated with subtype, histone mutation, and prognosis in HGG

**Fig. 5:**
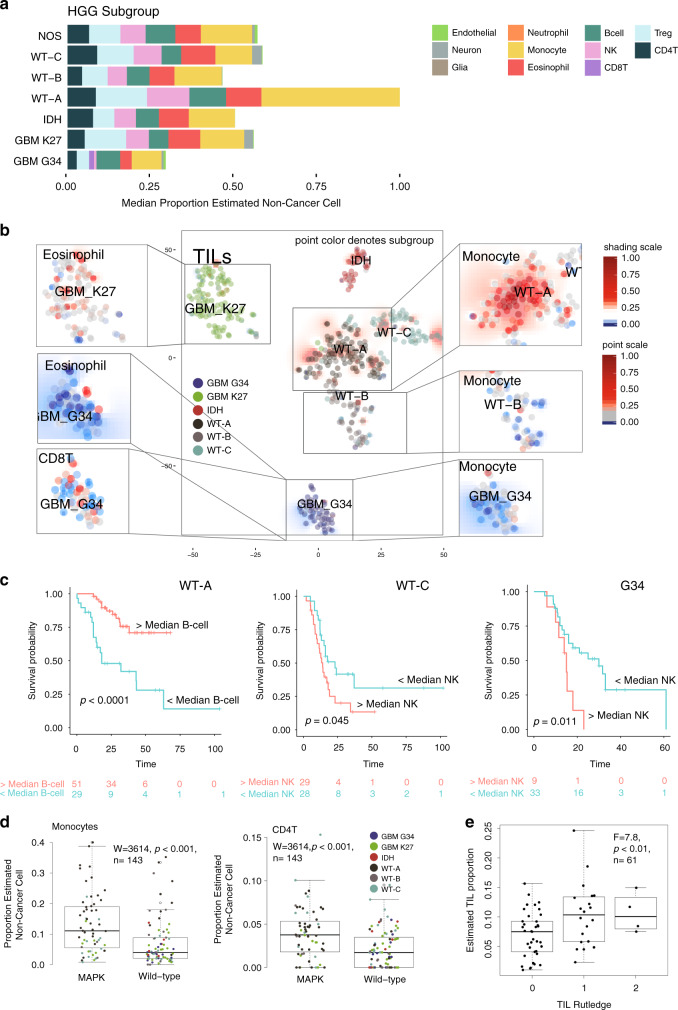
Deconvolution analysis of pHGG immune infiltration by subgroup. **a** Barplots of the estimated median infiltration of specific cell types as a proportion of all non-cancer cell types (range scaled from 0 to 1) in 401 pHGG (pediatric High-Grade Glioma) by subgroup (as per Mackay et al.^38^). **b** t-SNE plot representing the methylation profiles of 401 pHGG. The colors of dots in the central panel map to subgroup. Background shading represents the 2D spatial density estimation of the amount of tumor-infiltrating lymphocytes (TILs); red shading equals relatively greater than average infiltration and blue less than average. Exploded side panels represent enlarged areas of interest wherein both dot color and background shading represent the relative amount of the particular immune cell infiltration denoted. Red denotes relatively greater than average infiltration and blue less than average. **c** Kaplan–Meier plot showing significant difference in overall survival in WT-A (log-rank, *p* < 0.001, *n* = 80), WT-C (log-rank, *p* = 0.045, *n* = 80), and G34 subgroups (log-rank, *p* = 0.011, *n* = 42) by low (<median) or high (>median) levels of B-cell and NK infiltration. **d** Boxplot showing the proportion of monocytes and CD4T cells in pHGG by the presence/absence of a MAPK mutation (Wilcoxon = 3614, *p* < 0.001, *n* = 143). Box represents interquartile range, center line represents median, whiskers represent range of minima and maxima excluding outliers. **e** Boxplot showing TIL proportion as estimated by methylCIBERSORT for a subset of pHGG samples for which histopathology-based estimates of lymphocyte infiltration were available (*F* = 7.8, *p* = 0.007, *n* = 61). Patients are categorized as per Rutledge et al.^47^. Box represents interquartile range, center line represents median, whiskers represent range of minima and maxima. Estimates of TILs were significantly greater in patients classified as Categories 1 (present) or 2 (abundant) than Category 0 (absent).

Finally, we analyzed a cohort of 401 primarily pHGG samples published by Mackay et al.^38,39^. pHGG were, on average, infiltrated predominantly by monocytes (26% of non-cancer cells), Tregs (15%), and eosinophils (13%) (Fig. 5a). CD8T infiltration in pHGG was generally less than MB and MRT. Several cell types varied significantly with respect to tumor subgroup as defined by MacKay et al.^38^, i.e., WT-A, WT-B, WT-C, IDH, GBM G34, and GBM K27. These include monocytes, CD8T, TILs, and eosinophils (each *p* < 0.001) (see Supplementary Data 1 and Fig. 5a, b). Post hoc testing shows significantly greater monocytes in WT-A vs. other subgroups (2.9-fold, all comparisons *p* < 0.001), significantly greater CD8T in GBM with G34 mutations (1.7-fold, all comparisons *p* < 0.05), and significantly less eosinophils in GBM with G34 mutations (2.3-fold less, all comparisons *p* < 0.001). Furthermore, the number of TILs and indeed the overall level of immune cell infiltration is significantly higher in the WT-A subgroup (1.6-fold greater and 1.9-fold greater, respectively, all comparisons *p* < 0.01) and significantly lower in GBM G34 than other pHGGs (1.8-fold and 2.0-fold, respectively, all comparisons *p* < 0.01). The WT-A subgroup generally contains pHGG, otherwise referred to as PXA or LGG-like; they are also enriched for mitogen-activated protein kinase (MAPK) mutations (genes *NF1*, *FGFR1*, *NTRK2*, *BRAF*, see Supplementary Data 1).

Consensus clustering of pHGG immune cell estimates identifies an optimal three immune clusters referred to here as pHGG_IC1-3_, which cut across each of the pHGG subtypes (Supplementary Fig. 10A). pHGG_IC3_ overlaps primarily with panCNS_IC1_/panCNS_IC2_ and pHGG_IC2_ overlaps with panCNS_IC3_ (Supplementary Fig. 5C). pHGG_IC1_ constitutes 31% (126/401) of all pHGG and is characterized by high proportions of Tregs, eosinophils, NK, and CD4T. pHGG_IC2_ constitutes 17% (71/401) of all pHGG and is characterized by high proportions of monocytes and a disproportionately high frequency of WT-A subtypes; 77% (55/73) of all pHGG_IC2_ are also WT-A. pHGG_IC3_ constitutes 51% (204/401) of all pHGG and tumors show intermittently moderate levels of CD8T and relatively low levels of other infiltrating immune cell types. Eighty-seven percent (43/49) of all GBM G34 belong to this cluster (Supplementary Fig. 10A, B). As pHGG generally had higher monocytic infiltration than MB/MRT, we took the opportunity to consider what proportion of the monocyte signature might be attributed to microglia as opposed to infiltration from peripheral blood. In the absence of an appropriate methylation signature, we adapted a set of expression markers from Haage et al.^45^, which can distinguish peripheral monocytes from microglia. Where we possessed parallel expression profiles, we were able to estimate that a median 52% (range of 28–77%) of the monocyte infiltration could be attributed to microglia. This is consistent with proportion estimated by FACS in adult HGG as reported by Gabrusiewicz et al.^46^ (Supplementary Fig. 11).

Examining the association of cell infiltration with survival within each of the pHGG subgroups using Cox regression reveals the following significant associations (see Supplementary Data 1). Lower than median concentrations of B-cell and CD8T in WT-A patients are associated with a poor OS (HR = 4.3 CI95%^upper^ = 9.0, CI95%^lower^ = 2.0, *p* < 0.001, *n* = 80 and HR = 4.3, CI95%^upper^ = 18.2, CI95%^lower^ = 1.1, *p* = 0.047, *n* = 80, respectively). Higher than median concentrations of CD4T and NK in GBM G34 patients is associated with a poor OS (HR = 2.4, CI95%^upper^ = 5.3, CI95%^lower^ = 1.09, *p* = 0.028, *n* = 42 and HR = 3.0, CI95%^upper^ = 7.3, CI95%^lower^ = 1.2, *p* = 0.016, *n* = 42, respectively) (Fig. 5c and Supplementary Fig. 10C, D).

Clinico-pathological/biological features examined for association with estimated cell types include WHO stage, gender, age <1 year or age <3 years, and the presence of *BRAF* and/or other MAPK mutation. Several immune types were significantly associated with these clinico-pathological criteria (see Supplementary Data 1). As previously noted^39^, the presence of MAPK mutations was associated with higher immune cell infiltration, specifically of monocytes and CD4T cells (*W* = 3614 and *W* = 3453, respectively, both *p* < 0.001). In addition, nine patients who possessed a hypermutator phenotype showed a significantly higher estimated levels of TILs (KW = 5.0, *p* = 0.025, *n* = 137). For a subset of samples, histopathology-based estimates of lymphocyte infiltration were available, which categorized patients as per Rutledge et al.^47^. Estimates of TILs were significantly greater in patients classified as Categories 1 (present) or 2 (abundant) than Category 0 (absent) (*F* = 7.839, *p* < 0.01, *n* = 61). Again, taken as a whole, the significant relationships between molecular subgroup, prognosis, mutation, and immune infiltration in pHGG are clear.

## Discussion

Using a methylation-based deconvolution analysis, we have described the TIME of >6000 individual (primarily pediatric) CNS tumors. We find diversity in TIME composition across these CNS tumors and demonstrate significant associations variously with tumor type, subtype, stage, grade, location, mutation, and survival. The notion of the CNS, and by association CNS tumors, as immune privileged and inaccessible to immune cells is increasingly outdated^3^; nevertheless, our analysis lends weight to the idea of a diverse TIME across a wide range of CNS tumors. We establish here a base of knowledge by which future, more focused and in-depth investigations into the TIME of particular pediatric CNS tumor types may be directed.

The implications of our results are as follows. First, that the nature of immune cell content is associated with—but not exclusively dictated by—a particular tumor type or subtype. Second, that at least three broad CNS TIME subgroups strongly associated with tumor type and grade can be identified by clustering immune cell types, and that within individual tumor types (MB, ATRT, and pHGG) further immune subgroups may be described. Immune subgroups cut across the conventional CNS molecular tumor subgroups such that a patient may simultaneously belong to a given molecular subgroup and also independently a particular immune subgroup. Furthermore, these immune subgroups have different immunophenotypic characteristics (different CYT scores, expression of *PDL1*, etc.) and are associated with WHO Grade. Third, that key molecular features recognized as molecular drivers, such as *MYC* amplification in MB or *H3.3G34* mutations in HGG, are associated with distinct TIMEs and particular infiltrating cell types raising the possibility that these mutations are directly influencing the tumor microenvironment, perhaps as an adjunct to their intrinsic oncogenic mechanism. Fourth, that by extracting molecular information about TIME, we are able to access significant prognostic information independent of conventional molecular subgroups raising the possibility of their future incorporation into existing prognostic biomarker schemes. It should be noted that prognostic associations with immune cell infiltration appear to be context dependent; increased CD8T infiltration, for instance, does not universally denote a poor outcome. The most directly comparable experience in pHGG was the HERBY Phase II Trial^39^. High CD8+ infiltration was significantly associated with increased survival in 34 cases (of various subtypes) who received Temezolomide/radiotherapy and Bevacizumab for which our results in pHGG WT-A are in accordance. This is in contrast to ECRT where the opposite association is found with survival; this is a more unusual but not unprecedented finding, at least in other tumor types^48–50^.

Our results are broadly in accordance with the small number of recent investigations into immune infiltration in pediatric CNS tumors. Mackay et al.^39^ identified a relative lack of TILs in histone mutant pHGG compared to hypermutator and PXA-like (WT-A) subgroups, and this is borne out by our analysis here. Expression analysis of a mixed cohort of adult and pediatric gliomas by Bockmayr et al*.*^51^ identifies four immune clusters (including monocyte and T-cell-dominated clusters) not wholly inconsistent with our own. They show some associations with OS; however, these are mainly within the older (>40 years) and IDH-mutated subgroup. Bockmayr et al.^52^ also analyzed expression (by microarray) of immune markers in 763 MBs and concluded, like us, that MB_SHH_ tumors had larger numbers of T-cells overall than other subgroups. In contrast to our findings, they did not identify associations with MB survival as was the case for the study of 26 MB patients by Vermeulen et al.^53^.

methylCIBERSORT is a method of convenience, especially given the prevalent use of methylation profiling within pediatric CNS tumors. Limitations of tumor biopsies and representative sampling notwithstanding, our analysis provides much breadth but clearly not the depth that may be achieved by single-cell RNA-seq analysis. Our analysis is further limited by its reliance on pure cell populations and an assumption that the methylation signatures of these cells are identical to their counterparts within the tumor stroma. In other words, one cannot exclude the possibility that the tumor microenvironment could affect the immune cell methylome with respect to some of the CpGs within our signature matrix. It should also be noted that there is likely “dark-matter,” i.e., immune infiltration for which our reference population are absent or incomplete. Nevertheless, we have made efforts to validate and benchmark our estimates. First, by accurately estimating gold-standard flow-validated cell mixtures and by simulating mixtures of cell types (Fig. 1b, c). Second, by matching our own estimates of infiltrating lymphocytes and tumor purity with independent estimates or alternative algorithms (meTIL score), and by the use of control samples with known immune infiltration (Supplementary Fig. 4A–C). Third, by the use of parallel and independent expression data to match cytolytic score, TCR/BCR reads, and characteristic expression signatures with methylCIBERSORT estimates (Supplementary Figs. 6E, 7, and 8). Fourth, by the use of parallel histopathology/IHC-based estimates of infiltration in 30 MB/MRT samples and 61 pHGG (Figs. 1e, f and 5e).

**Fig. 6:**
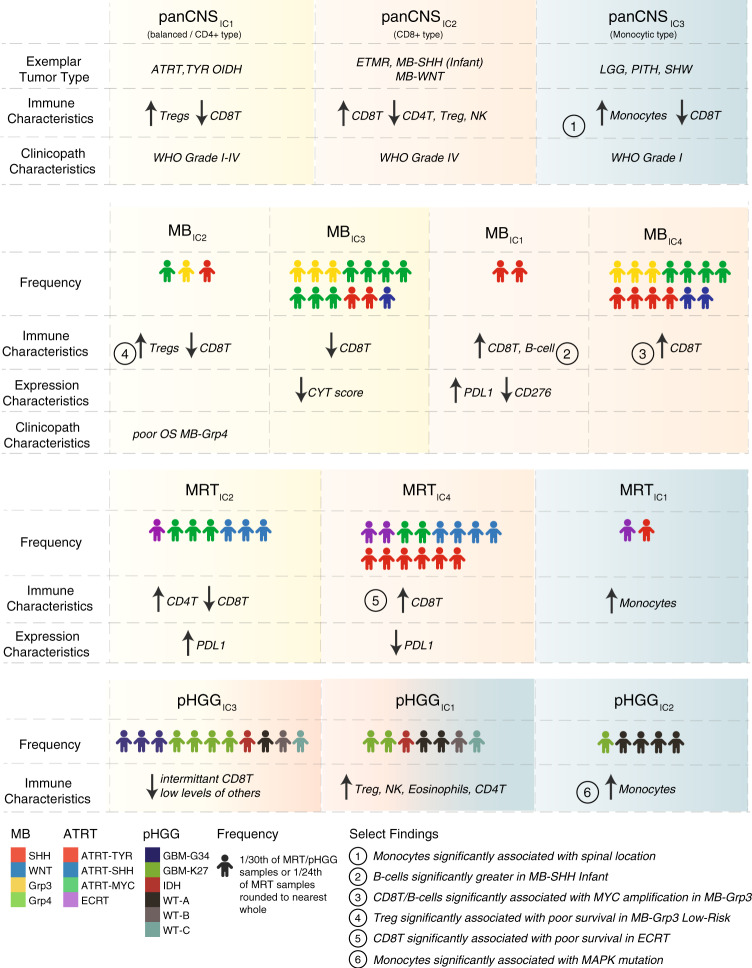
Summary of immune clusters and associated characteristics. **a** Schematic representation of the broadly defining features of immune clusters described in our analysis and a summary of select findings. Icons show the relative frequency of immune cluster membership and the distribution of particular subgroups. Color shading represents broad similarities between the panCNS immune clusters and the more refined tumor type-specific immune clusters. Yellow broadly denotes a pattern of relatively increased CD4+ T infiltration with low–moderate levels of infiltration of other cell types and a lack of CD8+ T, a “balanced/CD4+ type.” Red broadly denotes a pattern of relatively increased CD8+ T infiltration, a “CD8+ type,” and blue broadly denotes a pattern of relatively increased infiltration of monocytes, a “monocytic type”. MRT_IC3_ is not shown here, as it represents only a small number of patients.

Finally, our results and the immune clusters we have begun to develop here indicate important differences in TIME across pediatric brain tumor types; we summarize the key findings in Fig. 6. We can show that our immune clusters are clearly related to the expression of conventional immune targets such as *PDL1* in MB and ATRT, and in a broad sense indicate which immune subgroups are “hot” or “cold.” The immune clusters identified break down, broadly speaking, into the monocyte dominated (i.e., panCNS_IC3_ and pHGG_IC1/2_), the balanced, or CD4+ T-type (i.e., panCNS_IC1_, MB_IC2/3_, and MRT_IC2_) and the CD8+ T-type (i.e., panCNS_IC2_, MB_IC1/4_, and pHGG_IC3_). With such information, one may in future begin to match individuals or groups of individual TIMEs to immunotherapy responses or lack thereof. Even in the most simplistic terms, it seems to follow that an a priori paucity of infiltrating cytotoxic T-lymphocytes and the lack of a supportive TIME may be unconducive to immune checkpoint blockade as a therapeutic strategy, but instead may be amenable to approaches that alter the TIME or genetically redirect T-cell immunity.

In conclusion, this analysis gives first indications of the potential future therapeutic and prognostic possibilities of immuno-methylomic profiling as an adjunct to methylation/expression-based sub-classification. A future, in-depth, high-resolution approach incorporating spatial information is now required and we suggest that in silico deconvolution approaches may ultimately be used to triage and to inform selection of immunotherapy approaches in pediatric CNS tumor patients.

## Methods

### Construction of signature matrix for deconvolution

Raw 450K/850K Illumina Methylation array (.idat) files were obtained from the following sources and included reference samples additional to those used by Chakravarthy et al.^40^. CD8+ T-cells, CD14+ monocytes, CD19+ B-cells, and CD56+ NK profiles were obtained from the FlowSorted.Blood.450k & FlowSorted.CordBloodNorway.450k packages (R/Bioconductor); CD4+ T-cells and CD4+ Treg profiles were obtained from Gene Expression Omnibus (GEO) Dataset GSE49667; neuron and glial profiles from GSE50798; and endothelial profiles from GSE82234. One hundred and sixty-nine profiles distinguished between 12 broad cell types, namely effector T-cells (CD4+ CD45RA+ CD45RO− CD25−), regulatory T-cells (CD4+ CD45RA+ CD45RO− CD25+ FOXP3+), CD8 T-cells (CD8+), B-cells (CD19+), NK cells (CD56+), eosinophils (Singlec-8+ CCR3+), neutrophils (CD3- CD16+), monocytes (CD14+), endothelial cells (Huvec), glial cells (NeuN−), neuronal cells (NeuN+), and cancer (for full details see Supplementary Table 1). The signature matrix construction method and the majority of reference profiles were broadly as per Chakravarthy et al.^40^ with minor methodological changes. Specifically, the custom limma-based package (MethylCIBERSORT) developed by Chakravarthy et al.^40^ was used to fit linear models for each pairwise comparison between cell types with the following change made to the internal R function “MethylCIBERSORT::FeatureSelect.V4” for the purpose of this analysis with respect to the parameter “MaxDMPs.” Previously, the function selected *n* = MaxDMPs meaningful probes from the pairwise comparisons using a descending rank ordered by beta–delta, selecting only from the positively enriched probes. We amended the function to select MaxDMPs/2 unique probes from the positive- and negative-ranked beta–delta lists, respectively. This corrected a bias in the resulting signature matrix where the order of the pairwise comparisons had an effect on the resulting probes selected.

.idat files were processed, QC (Quality Control) checked, and normalized by single-sample Noob using the minfi package (R/Bioconductor). The custom limma-based function as described by Chakravarthy et al.^40^ was used to fit linear models performing a pairwise comparison between each of the cell types. A maximum of 200 top features per pairwise comparison were selected, restricting to probes showing a median *β*-value difference of 0.2 and false discovery rate of 0.01. *β*-Values were scaled to between 0 and 100, and probe means per cell type calculated to form a signature matrix compatible with CIBERSORT. The final signature matrix was selected from several matrices following a parameter search with benchmarking for deconvolution performance. Multiple cut-off parameters for the signature matrix were tested. Each reasonable combination of median *β*-value delta (0.2 and 0.3) and number of CpGs per pairwise comparison (100, 200, 300, 400, and 500) were tested using the known flow cytometry dataset (GSE112618) and mean methylCIBERSORT correlation, root-mean-square error scores, and the proportion of results not attributed to the six input populations were used as a measure of signature performance (Supplementary Fig. 1).

### Post-feature correction and QC checks

Variation in *β*-values in the CpGs identified by feature selection was noted for cell line profiles comprising the “Cancer” fraction. To prevent the estimation being confounded by cell line variability, post-selection filtering was applied on the signature matrix. Probes, where an average *β*-value difference between MB and MRT cell lines exceeded 0.1, with an SD exceeding 0.15 in MB cell lines, were removed from the final signature matrix. In addition, SD and variance were calculated probewise for all signature probes using the panCNS dataset. A per tumor type average SD threshold of 0.005 was applied to check for extremely low variation or invariant probes, which may be confounding or unlikely to represent real-world CNS tumor infiltration. Two sets of invariant probes were identified using this threshold; however, the first was clearly associated with a common neuronal, glial, or endothelial signature and the second was associated with a common cancer or immune cell-type signature. We therefore elected to retain these, as they were nevertheless clearly related to cell type and likely less variable as a result of relatively small levels of infiltration particularly of the neuronal, glial, and endothelial cell types. We did not identify any further probes, which were below the SD threshold. Furthermore, the mean and SD of *β*-values (for CpGs within the signature matrix) for each cell type and CNS tumor type were examined (see Supplementary Fig. 2A). The pattern of large *β*-value variations within the reference cell types and small variations between the CNS tumor types is consistent with these CpGs correctly representing a minor infiltrating population within a uniform majority cancer cell population.

### methylCIBERSORT

Input methylation matrices were created by processing raw.idat files as per above. Data were sourced from published GEO and ArrayExpress datasets and 79 previously unpublished MRT methylation profiles from SMARCB1-negative patients (see Supplementary Table 2 and Supplementary Data 2). CIBERSORT was run in relative mode using the provided R script (https://cibersort.stanford.edu) using 1000 permutations without quantile normalization.

### Validation and benchmarking of signature matrix

The signature matrix was inspected to verify that each cell type was accounted for by specific hypo/hypermethylated CpGs and not unduly compromised by batch effects. Likewise, t-SNE (package Rtsne, R/Bioconductor) was used to visualize the cell-type specificity of the signature matrix. The mean and SD of signature matrix CpGs were inspected in each of the 80 CNS tumor methylation types represented in dataset GSE109381 to identify possible outlier or confounding effects between immune-cell-type-specific CpGs and tumor cell types (see above).

Deconvolution performance was benchmarked against 18 gold standards, i.e., 6× methylation profiles of PBMC mixtures with known flow cytometry and 12× mixtures of reference pure population DNA in known proportions (GSE112618). Performance was also benchmarked against simulated mixtures generated to contain known quantities of a given cell type. This was achieved by taking the mean *β*-value of each pure cell reference and applying a random uniform distribution such that each simulated mixture contained a fixed amount of a given cell type (100 simulations for each) and a fixed 75% cancer cell signature derived from relevant cancer cell reference lines (see Supplementary Table 1). Correlation with methylCIBERSORT estimates was tested by the Spearman’s rank method. meTIL score (an independent measure of T-lymphocyte infiltration based upon methylation status of five CpGs—cg20792833, cg23642747, cg12069309, cg20425130, and cg21554552—was calculated following the code provided in Jeschke et al.^41^.

### Generation of synthetic mixtures for benchmarking signature performance

*β*-Values used to derive the signature matrix were averaged to obtain a mean profile for each signature matrix population. For 100 simulations per 12 signature populations (1200 total simulated samples), set proportions for each population were used while the other 11 were randomly calculated from a uniform distribution function, such that the sum of proportions was equal to 1. To simulate a tumor sample, the proportions were then scaled by 0.25 and a set proportion of 0.75 was defined for the ‘Cancer’ population as modeled by cancer cell line profiles. These proportions were used in a weighted mean to generate the final simulated mixture profiles. methylCIBERSORT was run on these samples as in other analyses.

### Signature to distinguish microglia from peripheral monocytes

Three expression signatures were adapted from Haage et al.^45^ to try to approximate the relative contribution of microglia to the monocyte populations predicted by methylCIBERSORT. To define microglia, we used the following genes: ENSG00000181631 (*P2RY13*), ENSG00000169313 (*P2RY12*), ENSG00000171659 (*GPR34*), ENSG00000142583 (*SLC2A5*), ENSG00000116774 (*OLFML3*), and ENSG00000183160 (*TMEM119*). To define peripheral macrophages, we used the following: ENSG00000126218 (*F10*), ENSG00000132205 (*EMILIN2*), ENSG00000198734 (*F5*), ENSG00000125730 (*C3*), ENSG00000119125 (*GDA*), ENSG00000188404 (*SELL*), and ENSG00000257017 (*HP*). To define all monocytes of whichever type, we used the following genes: (ENSG00000169896 (*CD11B*) and ENSG00000081237 (*CD45*). Genes were originally selected by Haage et al.^45^, because their expression was high and relatively equivalent within the target cell populations. We tried weighting according to their average expression and standardizing to variation, and attempted to quantify using ssGSEA/GSVA. Ultimately, each method differed little from the averaged standardized expression of each of the genes and so for the sake of simplicity this was used. A ratio of microglia to peripheral monocytes signatures was used to approximate the contribution of microglia in 36 pHGG for which parallel RNA-seq and methylation profiles were available. The signature scores were each significantly correlated (*p* < 0.001) with the proportion of monocytes estimated by methylCIBERSORT (see Supplementary Fig. 3).

### Visualization and clinico-pathological associations

Visualizations were created using the ggplot2, survminer packages (R/ Bioconductor). Relative cell proportions were categorized as higher or lower than median for the dataset in question. Bilinear density estimators were calculated from t-SNE coordinates using Rtsne, akima (R/Bioconductor/CRAN). Associations with survival were assessed using Cox proportional hazards and/or log-rank test. Patients were stratified according to whether they had greater than or lower than median for a given infiltrating cell type. Differences in cell proportions by subgroup or clinico-pathological criteria were tested by KW test with post hoc testing (Dunn’s test). Analysis of variance (F) or Wilcoxon test (W) was used where appropriate and where indicated. Associations between immune subgroup and categorical, i.e., clinico-pathological, variables were tested by *χ*^2^-test. All correlations and associated significance tests used Spearman’s rank and all *p*-values were adjusted for multiple hypothesis testing using Benjamini–Hochberg. Consensus clustering of immune estimates was by *k*-means using ConsensusClusterPlus (R/Bioconductor) testing range of *k* = 2 to *k* = 6. A support vector machine classifier using e1071 (R/CRAN) was trained on immune clusters derived from the panCNS analysis and tested on the MB, MRT, and pHGG cohorts. MiXCR^44^ analysis was used to derive CDR3 sequence clonotypes. ssGSEA was performed using gsva^54^(R/Bioconductor) using the GSE22886 gene sets from within the C7 library of MSigDB. When visualizing the median proportion of estimated non-cancer cells using a barplot in Figs. 2a, 3a, 4a, and 5a, the median estimated proportion of each cell type except for “cancer” were plotted after scaling to the maximum total value such that the bars give both an impression of the proportion of the estimated cell types and also the total relative amounts of infiltrating cells.

### Validation of predicted immune cell infiltration

An IHC panel using antibodies for CD8 1 : 250 dilution (Ventana Medical Systems Confirm anti-CD8 SP57; catalog number 790-4460) and CD20 1 : 200 dilution (Ventana Medical Systems Confirm anti-CD20 L26; catalog number 760-2531) was applied to pre-mounted slides using the fully automated Ventana BenchMark XT IHC system and standard detection reagents ultraVIEW Universal DAB Detection kit (Ventana Medical Systems; catalog number 760-500), incorporating antigen retrieval with Ventana ultra cell conditioning 1 and a hematoxylin counterstain (Ventana Medical Systems: catalog number 950-224). Leica Aperio membrane v9 analysis algorithm (Aperio ImageScope V12.4.0.7018) was applied to the stained slides.

### Reporting summary

Further information on research design is available in the Nature Research Reporting Summary linked to this article.

## Supplementary information

Supplementary Information

Descriptions of Additional Supplementary Files

Supplementary Dataset 1

Supplementary Dataset 2

Reporting Summary

## Data Availability

Reference cell profiles are available as part of the packages methylCIBERSORT, FlowSorted.Blood.450k & FlowSorted.CordBloodNorway.450k (R/Bioconductor), and GEO datasets GSE82234, GSE50798, GSE49667, GSE112618, GSE110554, and GSE88824. For the tumor cohorts: GEO datasets GSE70460, GSE109381, GSE60274, GSE93646, GSE85212, and GSE130051; ArrayExpress datasets E-MTAB-5528, E-MTAB-5552, and E-MTAB-6708. PedCBioPortal datasets phgg_herby and phgg_jones_meta_2017. Full details are given in “Methods,” Supplementary Information, and Supplementary Data. All remaining relevant data are available in the article, Supplementary Information, or from the corresponding author upon reasonable request.

## Code availability

Code used to generate the signature matrix as well as the resulting signature matrix have been deposited in Github [https://github.com/dannlbol/mcibersort_scripts].
