# Supplementary material for: Pediatric pan-central nervous system tumor analysis of immune-cell infiltration identifies correlates of antitumor immunity
Source: Nat Commun. 2020 Aug 28;11:4324. doi: 10.1038/s41467-020-18070-y (PMC7455736; doi:10.1038/s41467-020-18070-y)
Supplement: Supplementary file 1 — Supplementary Information [file 41467_2020_18070_MOESM1_ESM.pdf]

Supplementary Information

Pediatric Pan-CNS Tumor Analysis of Immune-cell Infiltration Identifies Correlates of Antitumor Immunity

Grabovska et al

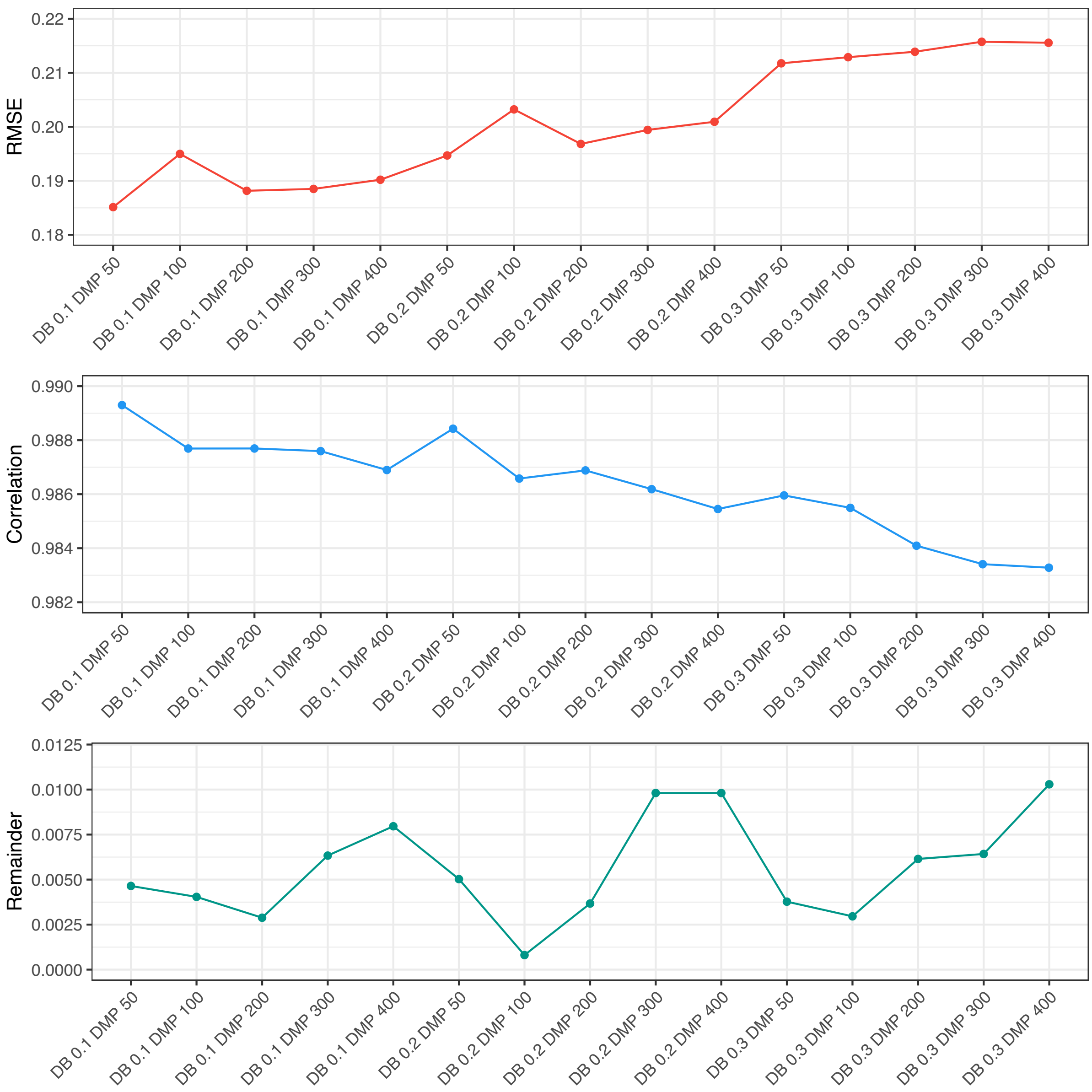

Supplementary Figure 1. Comparison of performance metrics for signature matrices. Performance metrics for methylCIBERSORT signature matrices given for a range of DMP (differentially methylated probes) and DB (delta beta) thresholds calculated using known flow-validated proportions in six control PBMC cell mixtures. Root mean square error (RMSE) and correlation are calculated as per Newman *et al*[1]. Remainder is defined as the total proportion of the methylCIBERSORT estimation result being assigned to populations of the signature matrix not found in the PBMC input mixtures. Processing time not shown, but higher DMP and DB parameters significantly increase signature matrix size and required computation time. Overall it should be noted that any changes in performance were relatively trivial.

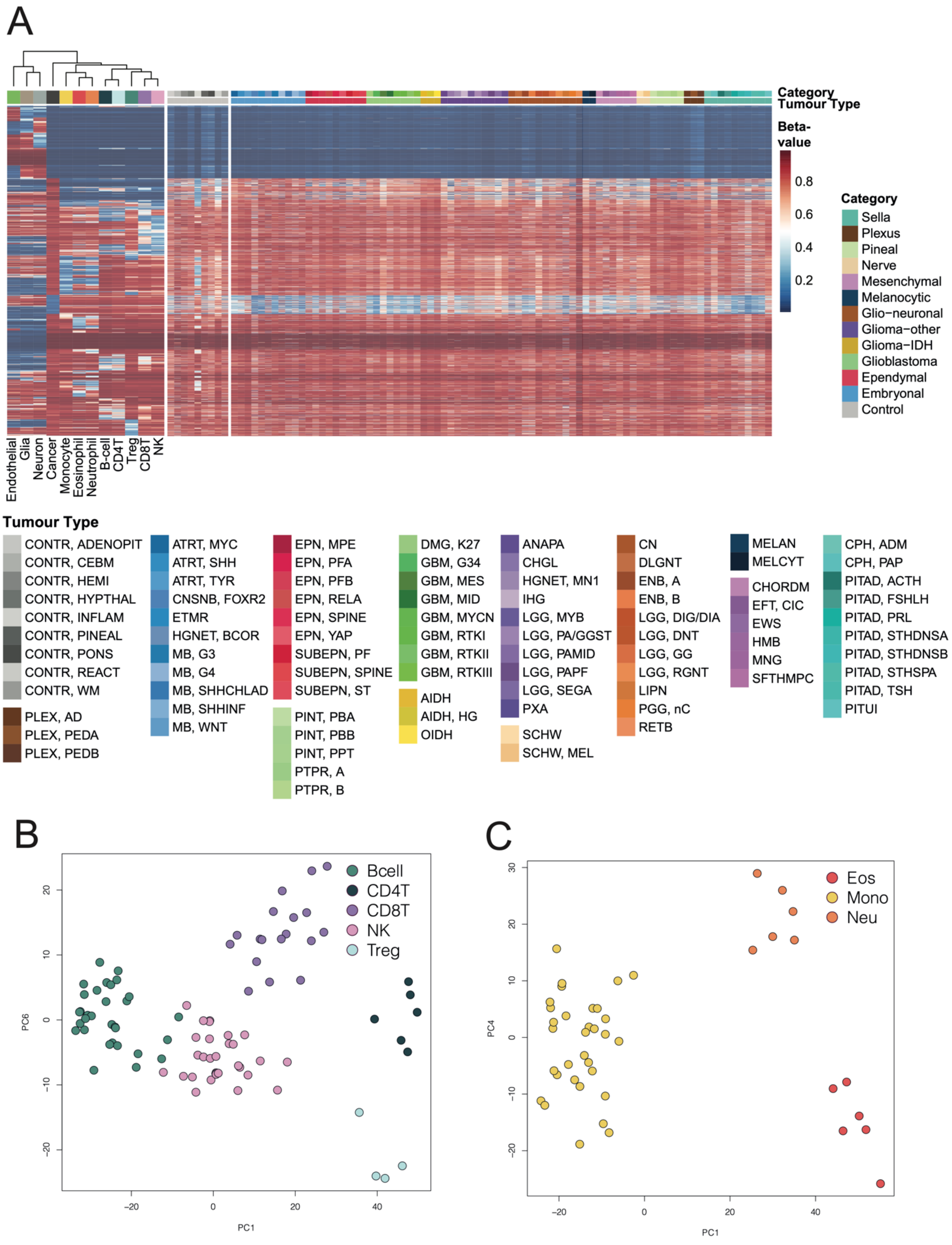

Supplementary Figure 2: Comparison of the signature matrix ability to differentiate between cell types. A. Heatmap showing mean beta values for CpGs (rows) used in the signature matrix by CNS tumor cell type/category for 3,763 CNS tumors and controls taken from Capper *et al*[2] demonstrating broad consistency of methylation across all CNS tumor types and independence of the signature from confounding tumor-type related effects. Left-hand side columns represent mean beta values for each reference cell type. CNS tumor types/subtype/category colors and abbreviations are as per Capper *et al*[2] full key is given in Supplementary Table 4. **B.** Principle Components Analysis showing the lymphocyte profiles used to create the signature matrix and **C.** the eosinophil, monocyte and neutrophil profiles.

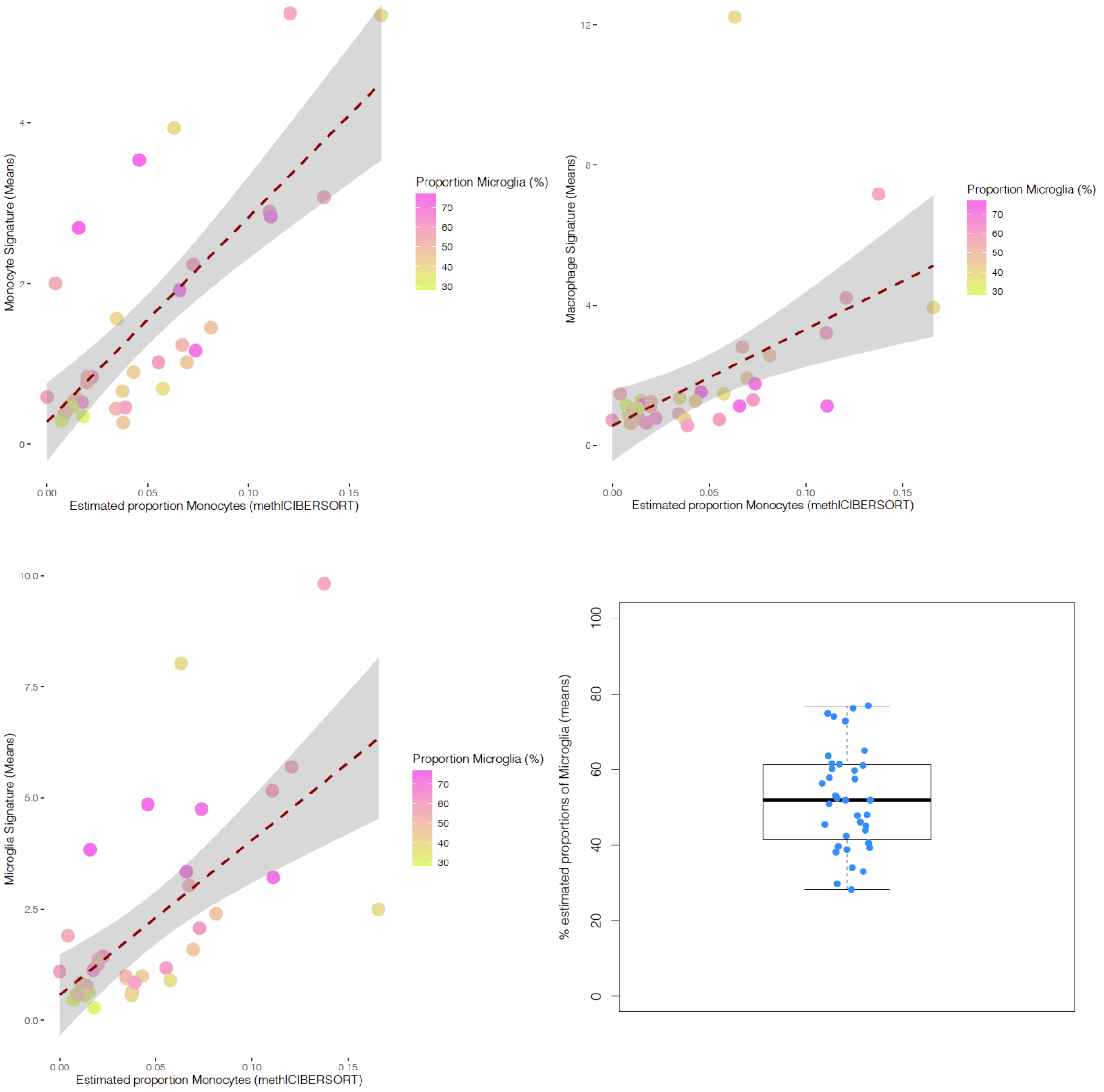

Supplementary Figure 3. Comparisons of estimated monocytes, macrophages and microglia infiltration. Scatterplot and significant correlation (each $p<0.001$) in 36 pHGG patients between estimated proportion of monocytes by methylCIBERSORT and expression signature scores defining all monocytes (top left), peripheral monocytes (top right) and microglia (bottom left). Heatmap scale shows the estimated proportion of the total monocyte expression signature attributable to microglia. Bottom right shows a boxplot of the estimated percentage of expression signature attributable to microglia in 36 pHGG patients.

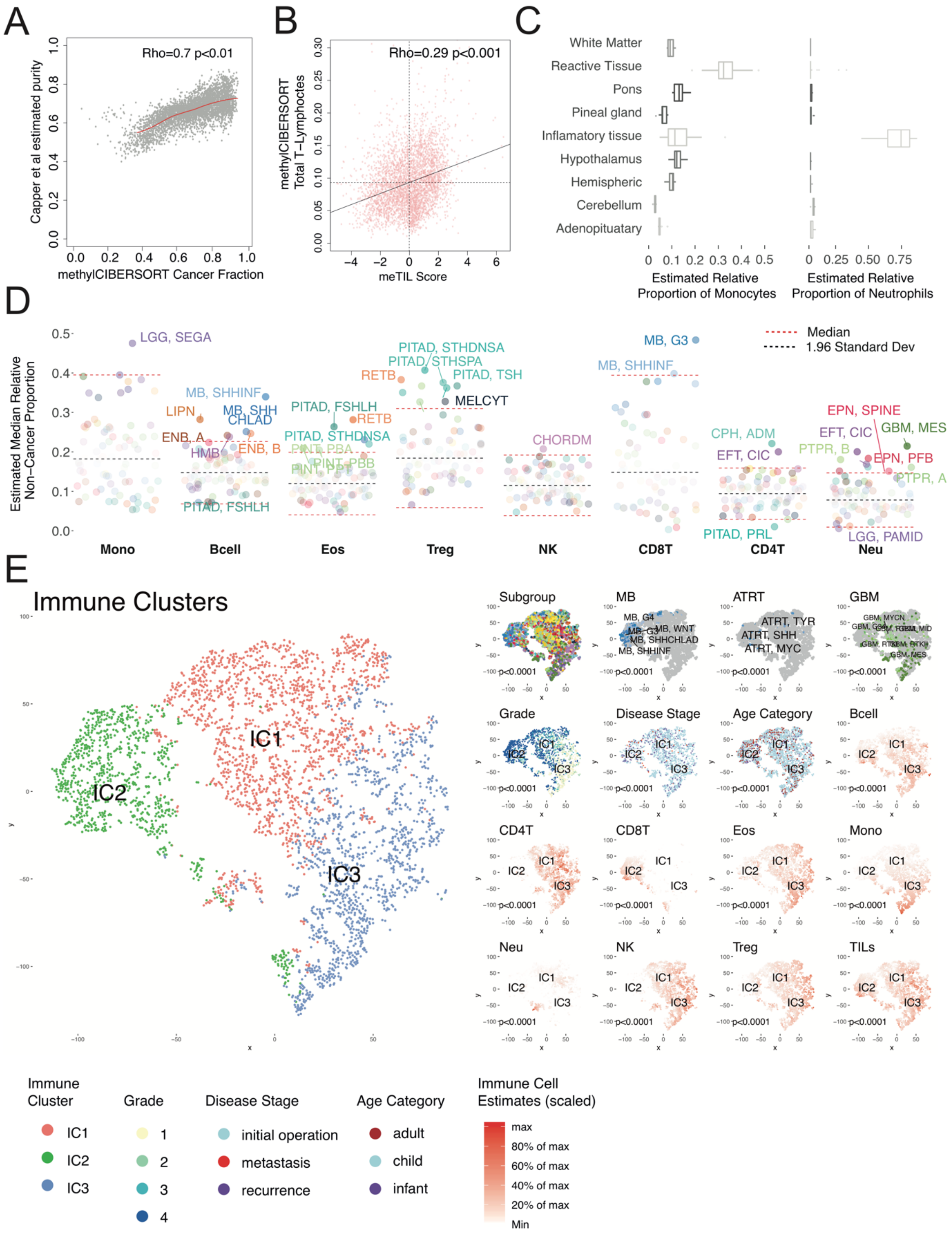

Supplementary Figure 4: Immune clustering of pan-CNS tumors and controls shows 3 distinct clusters. A. Scatterplot showing the estimated methylCIBERSORT cancer fraction correlates significantly with Capper *et al*'s[2] published estimates of tumor purity; Pearson's, Rho=0.7, p<0.001. **B.** Scatterplot showing the estimated methylCIBERSORT estimate of total T-lymphocyte infiltration correlates significantly with an independent meTIL score (as per Jeshcke *et al*[3]); Pearson's, Rho=0.29, p<0.0001. **C.** Boxplot showing methylCIBERSORT estimates of

monocyte and neutrophil infiltration in control samples included within the Capper *et al* [2] cohort; n=119 biologically independent samples. Box represents interquartile range, centre line represents median, whiskers represent range of minima and maxima excluding outliers. As expected significantly greater proportions of monocytes and neutrophils were observed in reactive and inflammatory tissues respectively **D.** Dotplots of the estimated median infiltration of specific cell types as a proportion of all non-cancer cell types by tumor type/subtype highlighting the range and variation of immune cell infiltration in different CNS tumor types. Dotted black lines represent the median for histological categories for a given cell type and dotted red lines mark a variance threshold of 1.96 standard deviations. Selected tumor types which exceed these variance thresholds are marked with text. **E.** t-SNE plot showing clustering of the panCNS cohort by immune cell estimates. Large panel shows three immune clusters (IC1-3), smaller panels show the location and distribution of tumors of particular subgroup, grade, stage and age, immune cell estimates are represented as a red-white color scale. P-values represent statistical test for non-random association of a given characteristic with immune-cluster.

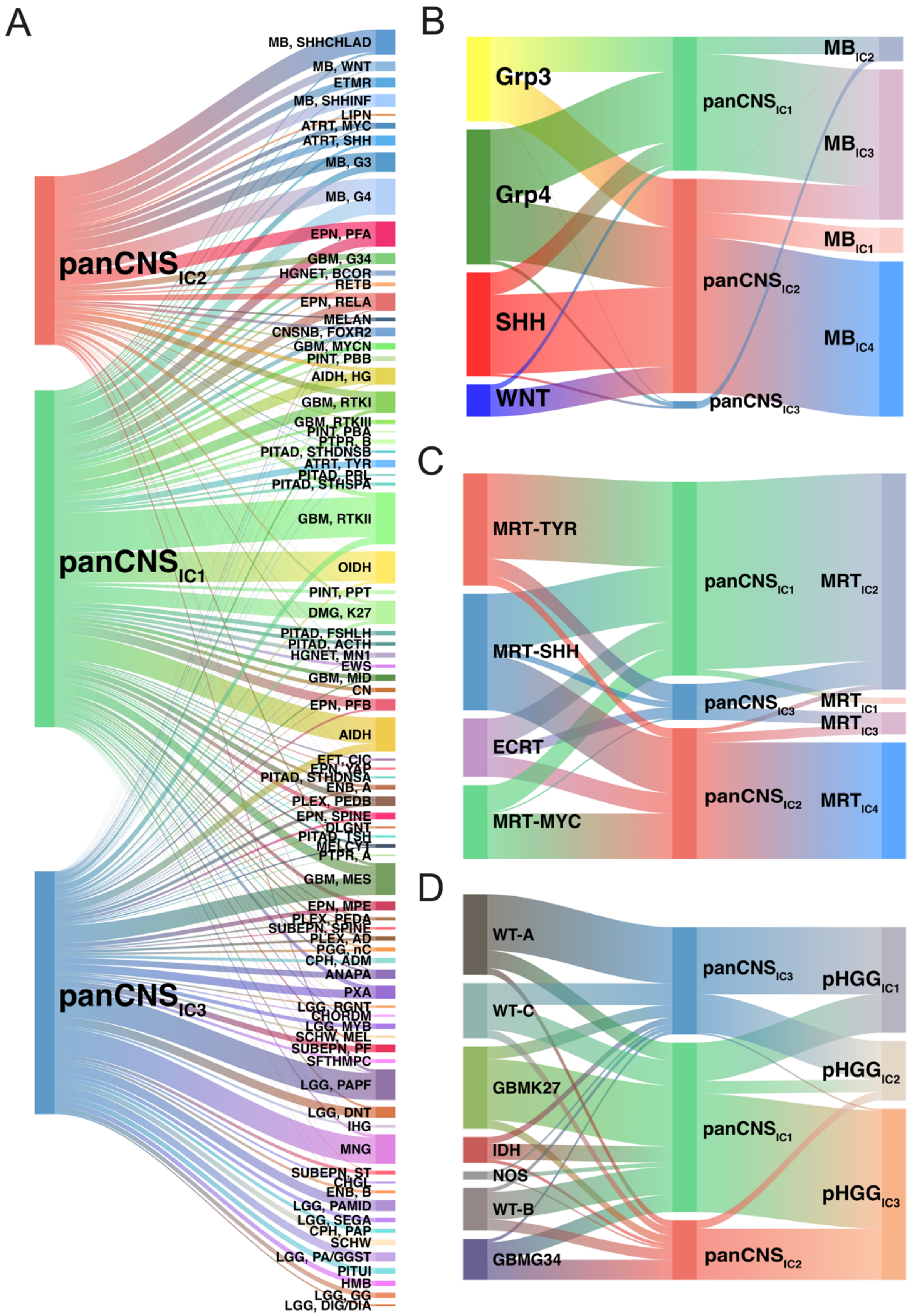

Supplementary Figure 5: Associations between immune cell infiltration clustering and molecular subgrouping. **A**. Sankey plot showing proportions shared between panCNS immune clusters and tumor subgroup. Colors and text label marking tumor type/subtype are as per Capper *et al*[2] (for full key see Supplementary Table 4). **B.** Sankey plot showing proportions shared between panCNS immune clusters, MB immune clusters and MB subgroup. **C.** Sankey plot showing proportions shared between panCNS immune clusters, MRT immune clusters and MRT subgroup. **D.** Sankey plot showing proportions shared between panCNS immune clusters, pHGG immune clusters and pHGG subgroup.

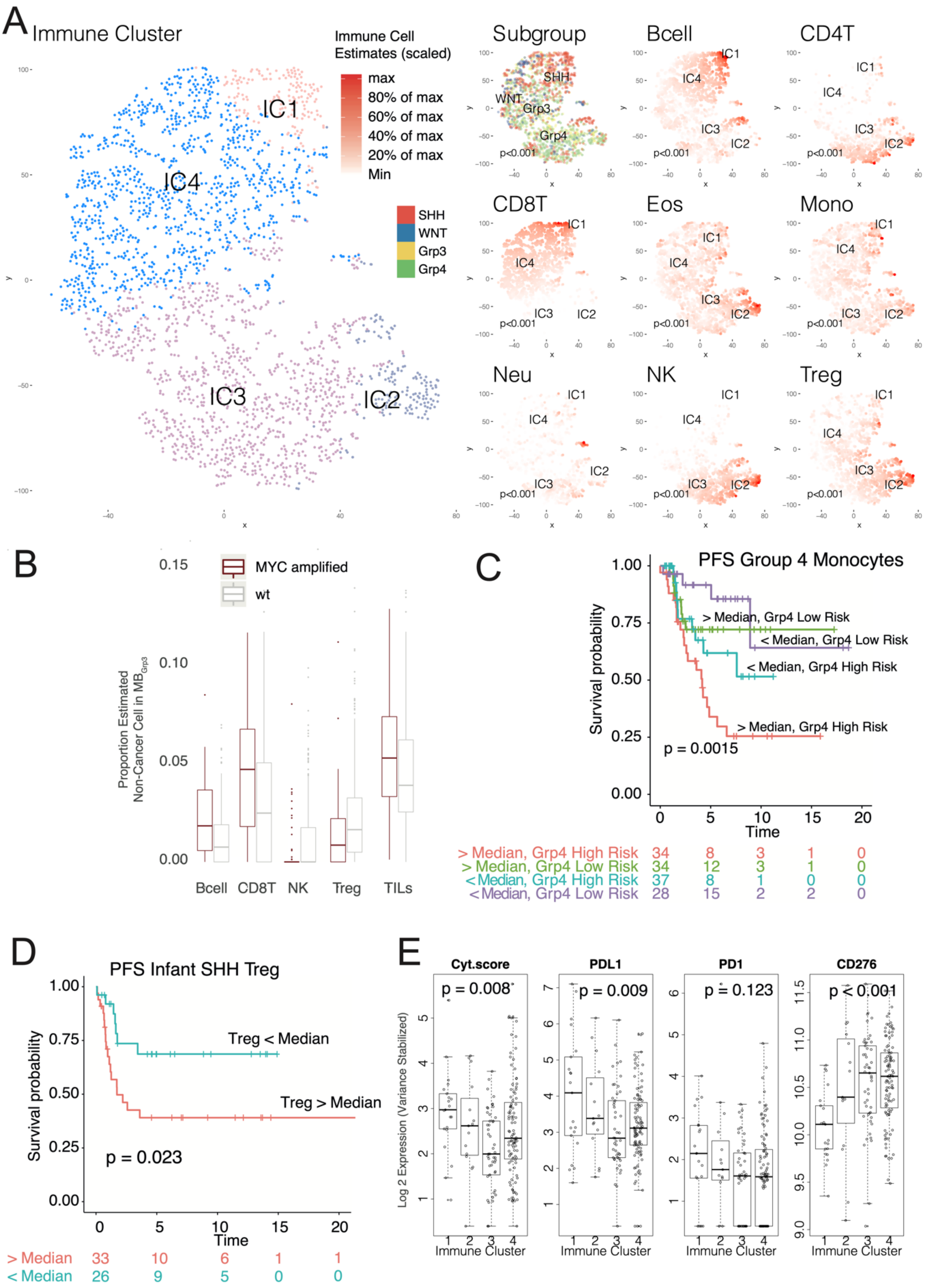

Supplementary Figure 6: Clustering of estimated immune cell infiltration in MB shows four clusters. A. t-SNE plot showing clustering of the MB cohort by immune cell estimates. Large panel shows four immune clusters (MB_{IC1-4}), smaller panels show the location and distribution of tumor of particular subgroup, immune cell estimates are represented as a red-white color scale. P-values represent statistical test for non-random association of a given

characteristic with immune-cluster. **B.** Boxplot showing proportion of non-cancer cells by presence of MYC amplification in MB_{Grp3}; n=408 biologically independent samples. Box represents interquartile range, centre line represents median, whiskers represent range of minima and maxima excluding outliers. **C.** Kaplan-Meier plot showing significantly different progression free survival (PFS) in MBGrp4 by low (< median) or high (> median) levels of monocyte infiltration; Log-Rank, p=0.0015, n=133. **D.** Kaplan-Meier plot showing significantly different progression free survival (PFS) in infant MB_{SHH} by low (< median) or high (> median) levels of Treg infiltration; Log-Rank, p=0.023, n=59. **E.** Boxplot showing expression of *PDL1* (F=4.0, p=0.009), *PD1* (F=1.9, p=0.123), *CD276* (F=6.251, p=0.000457) and CYT score (Cyt.score F=4.1, p=0.008), by MB immune cluster; n=185 biologically independent samples. Box represents interquartile range, centre line represents median, whiskers represent range of minima and maxima excluding outliers.

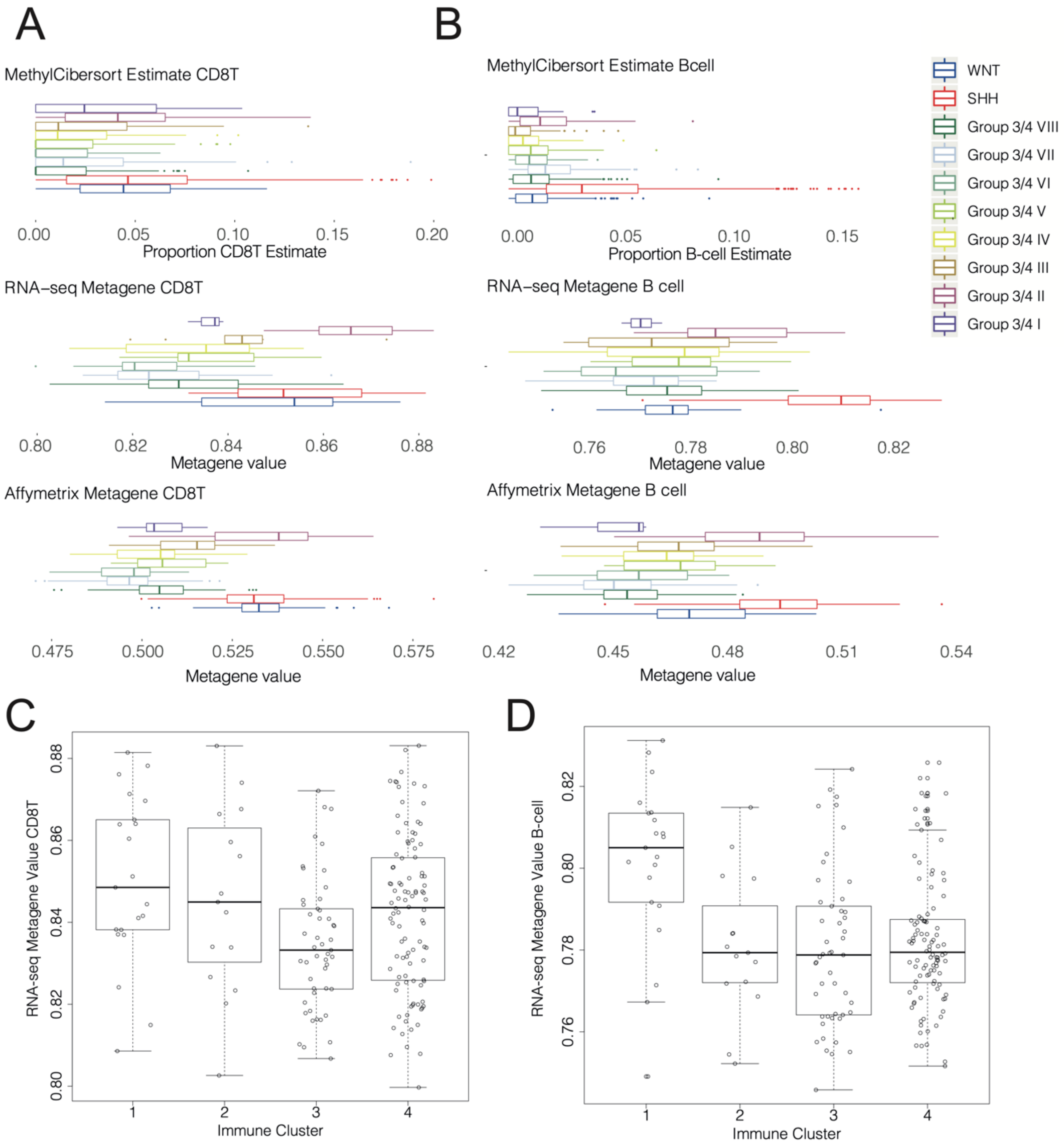

Supplementary Figure 7: Comparisons of methylation estimates of immune infiltration with parallel RNA-seq. A. Boxplot showing methylCIBERSORT estimates of CD8T infiltration and ssGSEA estimates of CD8T expression signature and **B**. B-cell expression signature in n=185 biologically independent parallel RNA-seq and n=763 biologically independent Affymetrix expression array data by Medulloblastoma subtype. **C.** Boxplot showing ssGSEA estimates of CD8T expression signature and **D**. B-cell expression signature in n=185 biologically independent parallel RNA-seq and n=763 biologically independent Affymetrix expression array profiles by immune cluster. All boxes represent interquartile range, centre line represents median, whiskers represent range of minima and maxima excluding outliers.

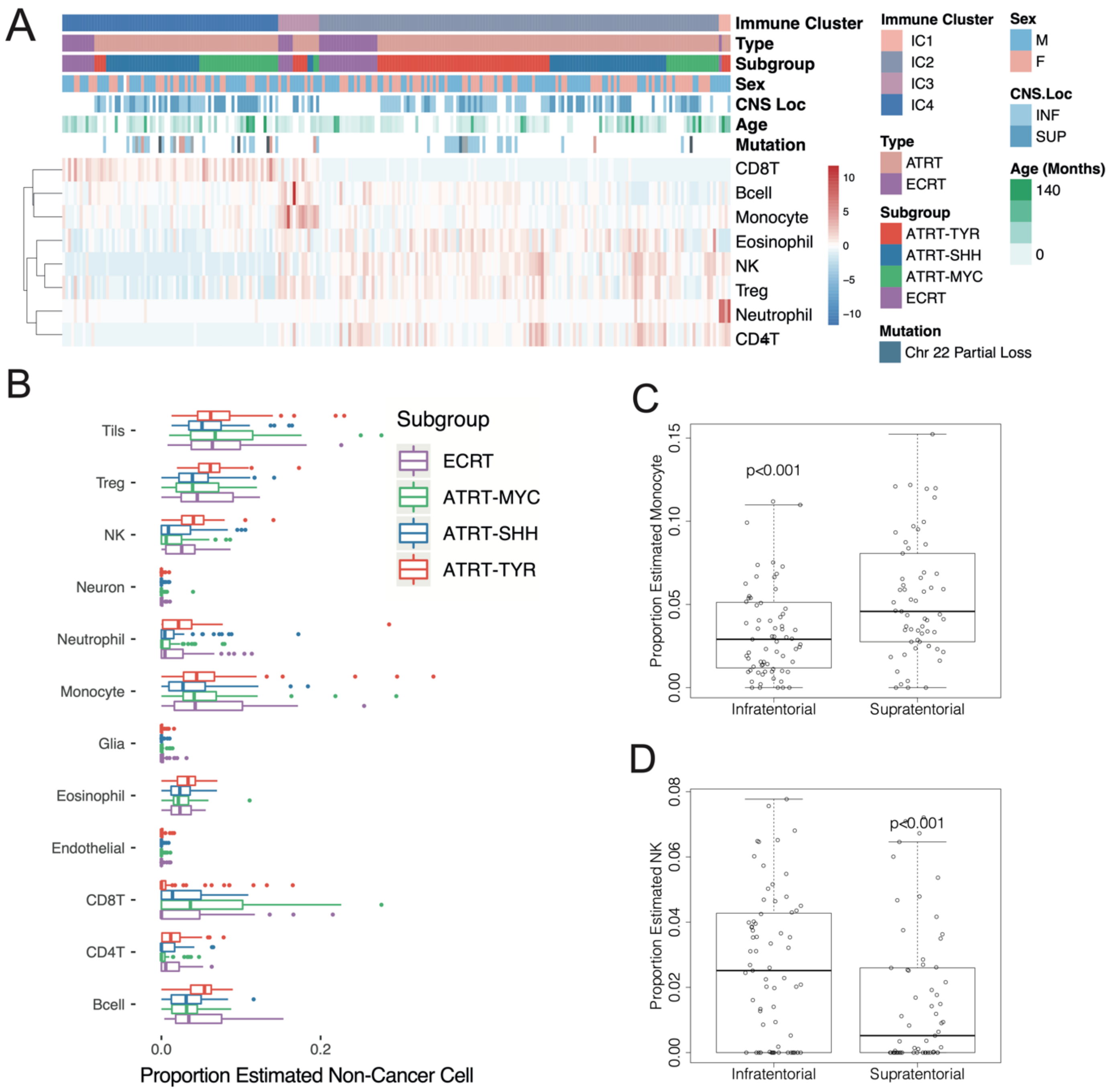

Supplementary Figure 8: Clustering of estimated immune cell infiltration of MRT and associated subgroup. A. Heatmap showing row-scaled relative levels of immune cell infiltration in 229 Malignant Rhabdoid Tumors (MRT) ordered by immune cluster MRT_{IC1-4}. **B**. Boxplot showing estimated proportion of cell infiltration by molecular subgroup; n=229 biologically independent samples. **C.** Boxplot showing estimated monocyte infiltration W = 1469.5, p-value = 0.001 n=132 and **D.** NK infiltration in ATRT by CNS location (infratentorial/supratentorial) ; W = 2726.5, p-value = 0.009, n=132 biologically independent samples. All boxes represent interquartile range, centre line represents median, whiskers represent range of minima and maxima excluding outliers.

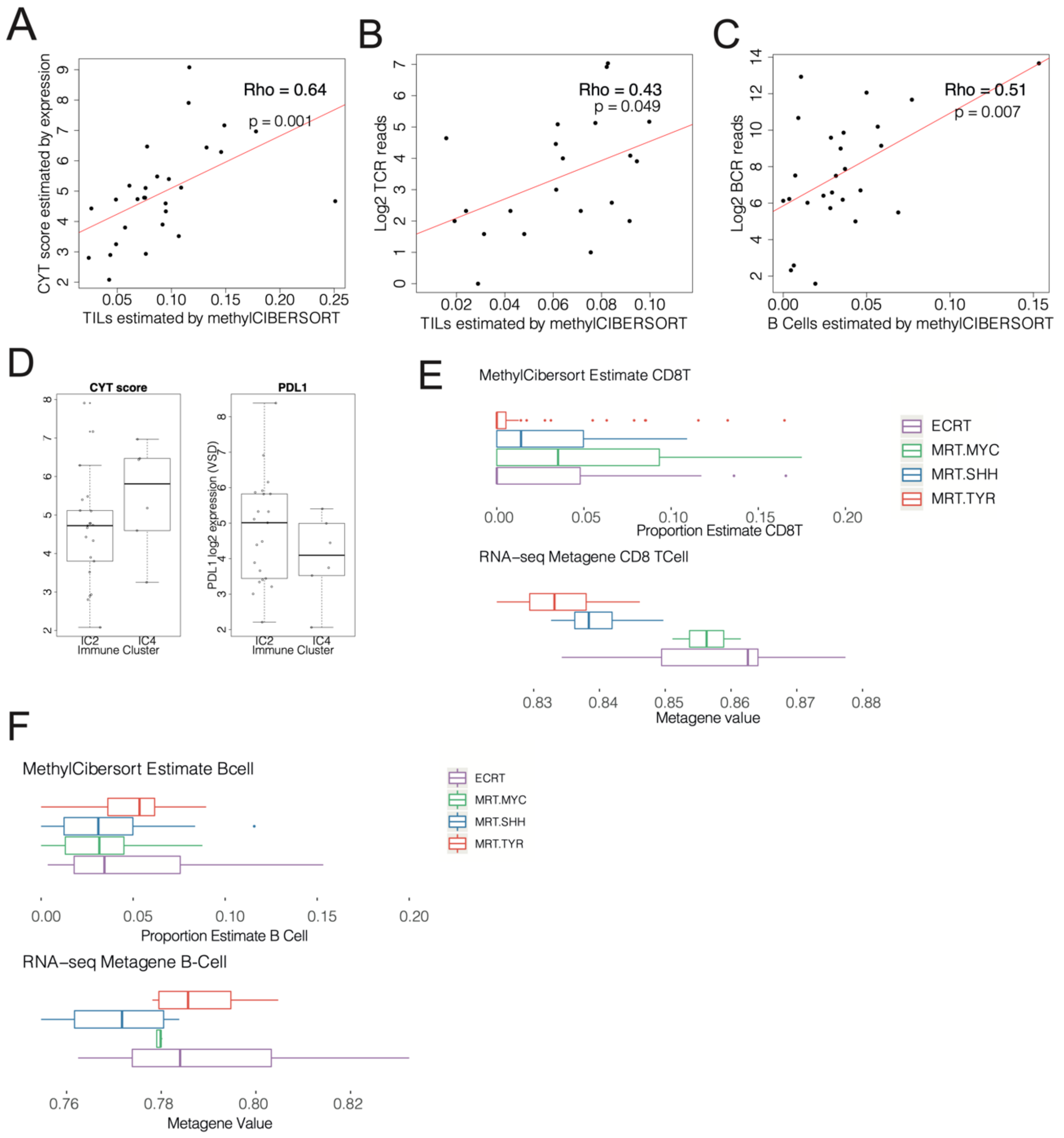

Supplementary Figure 9: Comparisons of methylation and expression estimates of immune infiltration in MRT. A. Scatterplot showing CYT score correlation in Malignant Rhabdoid Tumors (MRT) with proportion of TILs as estimated by methylCIBERSORT (Pearson, Rho = 0.64, p =0.001). **B.** scatterplot showing correlation of normalized CDR3 TCR reads with proportion of T-lymphocytes estimated by methylCIBERSORT (Pearson, Rho = 0.43, p=0.049) and **C.** BCR reads with proportion of B cells estimated by methylCIBERSORT (Pearson, Rho = 0.51, p=0.007). **D.** Boxplot showing CYT score and PDL1 expression by MRT immune cluster; n=28 biologically independent samples. **E.** Boxplot showing methylCIBERSORT estimates of CD8T infiltration and ssGSEA estimates of CD8T expression signature and **F.** B-cell expression signature in n=28 parallel RNA-seq profiles by MRT subtype. All boxes represent interquartile range, centre line represents median, whiskers represent range of minima and maxima excluding outliers.

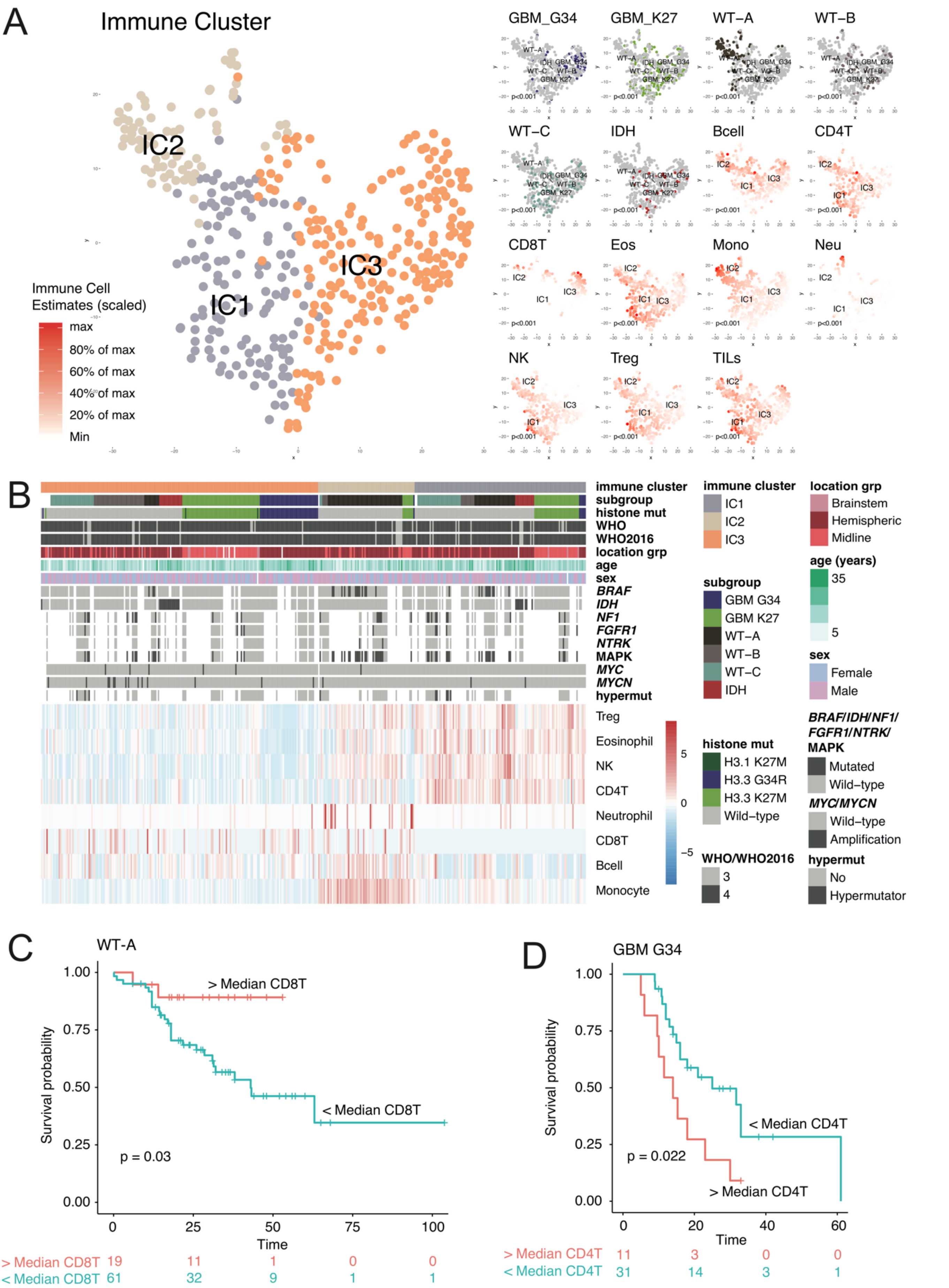

Supplementary Figure 10: Clusters of immune cell infiltration estimates in pHGG and associated characteristics. A. t-SNE plot showing clustering of the pHGG cohort by immune cell estimate large panel shows four immune clusters ($pHGG_{IC1-3}$), smaller panels show the location and distribution of tumors of particular subgroup, immune cell estimates

are represented as a red-white color scale. P-values represent statistical test for non-random association of a given characteristic with immune-cluster. **B.** Heatmap showing row-scaled relative levels of immune cell infiltration in 401 pHGG ordered by immune cluster $pHGG_{IC1-3}$. **C.** Kaplan-Meier plot showing significant difference in overall survival in WT-A patients by low (< median) or high (> median) levels of CD8+T infiltration; Log-Rank, p=0.03, n=80 biologically independent samples. **D.** Kaplan-Meier plot showing significant difference in overall survival in G34 patients by low (< median) or high (> median) levels of CD4T infiltration; Log-Rank, p=0.022, n=42 biologically independent samples.

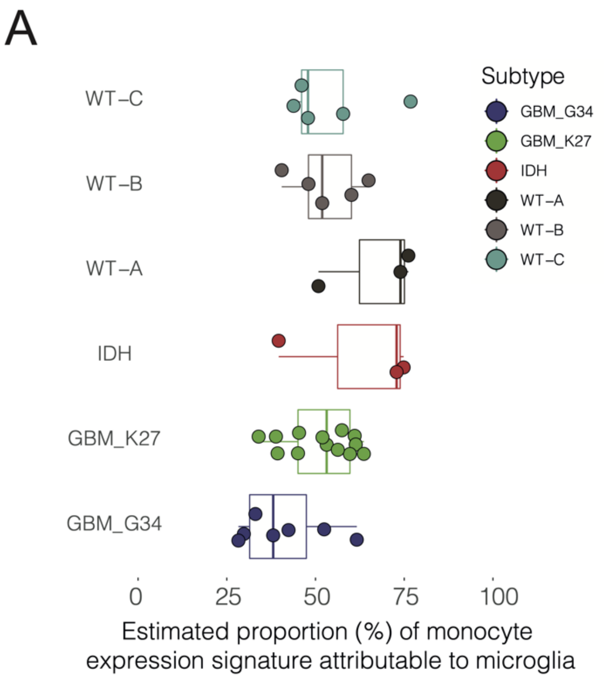

Supplementary Figure 11: Estimated proportions of monocyte signature attributable to microglia in pHGG. A. Boxplot showing the estimated proportion of the total monocyte expression signature attributable to microglia in 36 biologically independent pHGG samples by subgroup. Boxes represent interquartile range, centre line represents median, whiskers represent range of minima and maxima excluding outliers.

Supplementary Table 1. Data source, publication and short description of the isolation method used for samples used to create the reference signature matrix.

Dataset	Cell Type (number)	Isolation method
PMID: 27785870 [4]; GSE82234 (GEO/NCBI)	Endothelial/HUVEC (6)	Human umbilical vein endothelial cell donors; Supplemented Endopan 3 medium culture
Provided by Ankur Chakravarthy<br>PMID: 24057217 [5]; GSE50798 (GEO/NCBI)	Glia (12)	Dissected Medial Orbito-Frontal Cortex tissue; NeuN− FACS (FACS Vantage with DiVa)
	Neuron (12)	Dissected Medial Orbito-Frontal Cortex tissue; NeuN+ FACS (FACS Vantage with DiVa)
Provided by Ankur Chakravarthy<br>PMID: 23974203 [6]; GSE49667 (GEO/NCBI)	CD4+ T Effector (6)	Healthy donor blood buffy coat samples; CD45RA+ CD45RO− CD25− FACS
	CD4+ Treg (4)	Healthy donor blood buffy coat samples; CD45RA+ CD45RO− CD25+ FACS
FlowSorted.Blood.450k (Bioconductor)<br>PMID: 22848472 [7]	CD19+ B cells (6)	Healthy donor PBMC; Ficoll-Paque Plus - PBMC (GE Healthcare, Sweden); CD19+ MACS (Miltenyi Biotech, Germany)
	CD8+ T cells (6)	Healthy donor PBMC; Ficoll-Paque Plus - PBMC (GE Healthcare, Sweden); CD8+ MACS (Miltenyi Biotech, Germany)
	Eosinophil (6)	Healthy donor PBMC; Ficoll-Paque Plus - Granulocyte (GE Healthcare, Sweden); Eosinophil Isolation kit II (Miltenyi Biotech, Germany)
	CD14+ Monocyte (6)	Healthy donor PBMC; Ficoll-Paque Plus - PBMC (GE Healthcare, Sweden); CD14+ MACS (Miltenyi Biotech, Germany)
	Neutrophil (6)	Healthy donor PBMC; Ficoll-Paque Plus - Granulocyte (GE Healthcare, Sweden); CD16+ MACS (Miltenyi Biotech, Germany)
	CD56+ NK cells (6)	Healthy donor PBMC; Ficoll-Paque Plus - PBMC (GE Healthcare, Sweden); CD56+ MACS (Miltenyi Biotech, Germany)
FlowSorted.CordBlood.450k (Bioconductor)<br>PMID: 27019159 [8]	CD19+ B cells (14)	Healthy donor CBMC; Ficoll 1077; CD19+ MACS (Miltenyi Biotech, Germany)
	CD8+ T cells (12)	Healthy donor CBMC; Ficoll 1077; CD8+ MACS (Miltenyi Biotech, Germany)
	CD14+ Monocyte (15)	Healthy donor CBMC; Ficoll 1077; CD14+ MACS (Miltenyi Biotech, Germany)
	CD56+ NK cells (13)	Healthy donor CBMC; Ficoll 1077; CD56+ MACS (Miltenyi Biotech, Germany)
FlowSorted.CordBloodNorway.450k (Bioconductor)<br>PMID: 27494297 [9]	CD19+ B cells (11)	Healthy donor CBMC; Lymphoprep (Stem Cell Technologies, Norway); CD19+ FACS (BD FACS Aria)
	CD14+ Monocyte (11)	Healthy donor CBMC; Lymphoprep (Stem Cell Technologies, Norway); CD14+ FACS (BD FACS Aria)
	CD56+ NK cells (11)	Healthy donor CBMC; Lymphoprep (Stem Cell Technologies, Norway); CD56+ FACS (BD FACS Aria)
	Cancer (25)	MRT/MB Cell lines (D283, D341, D384, D425 x2, D458 x2, D556, DAOY, HDMB03, MED1, MED8A, ONS76, UW228, A204, BT12 x2, BT16, CHLA259, CHLA266 x2, G401 x2, WT1 x2)

Supplementary Table 2. Sources of the data used for CNS tumor cohorts.

**Dataset**	**Tumour Type (number)**
GSE70460 (GEO/NCBI)	ATRT (150)
GSE109381 (GEO/NCBI)	Multiple CNS malignancies (3905)
GSE60274 (GEO/NCBI)	Glioblastoma (68)
E-MTAB-5528 (n = 99), E-MTAB-5552 (ArrayExpress)/ PMID:29763623 (n = 71)[10], PMID:28966033 (n = 225)[11]. Additional (n = 6) (see Supplementary Data 2). Expression profiles (n=36) from pedcBioPortal (pedcbioportal.org) (PMID:28966033)[11]	Paediatric HGG/DIPG (401) *
E-MTAB-6708	MRT Primary (79)
PMID:28726821 [12], GSE93646, GSE85212, GSE130051 (GEO/NCBI) (see Supplementary Data 2).	Medulloblastoma (2325)

* When considering if a patient bore a MAPK pathway mutation the following genes were considered NF1(truncating frameshift/nonsense, disrupting translocation or predicted damaging missense), FGFR1 (known activating hotspot mutation), NTRK2 (translocation or tandem duplication of kinase domains), and BRAF (V600E). Mutations were either taken from or as per method described in Mackay *et al*[11].

Supplementary Table 3. Data used for independent validation, and with known flow cytometry measures.

Dataset	Cell Type (number)
GSE112618 (GEO/NCBI)	WBC mix (6)
GSE110554 (GEO/NCBI)	Artificial DNA mix: CD4+ T, CD8+ T, CD14+ monocyte, neutrophil, CD19+ B, CD56+ NK (12)
GSE88824 (GEO/NCBI)	CD19+ B cells (8)<br>CD4+ T (8)<br>CD8+ T cells (8)<br>CD14+ Monocyte (8)<br>neutrophil (8)<br>CD56+ NK cells (8) *

Supplementary Table 4. CNS tumor type abbreviations as given in Capper *et al*[2] and used within our manuscript is as follows.

Methylation Class Name	Methylation Class Name Abbreviated	Figure Colour Code
methylation class embryonal tumor with multilayered rosettes	ETMR	1693d6
methylation class medulloblastoma, WNT	MB, WNT	6da7d9
methylation class diffuse midline glioma H3 K27M mutant	DMG, K27	a3f187
methylation class central neurocytoma	CN	b85400
methylation class diffuse leptomeningeal glioneuronal tumor	DLGNT	d1723a
methylation class cerebellar liponeurocytoma	LIPN	de7b2c
methylation class low grade glioma, desmoplastic infantile astrocytoma / ganglioglioma	LGG, DIG/DIA	cd4d00
methylation class low grade glioma, dysembryoplastic neuroepithelial tumor	LGG, DNT	cb5216
methylation class low grade glioma, rosette forming glioneuronal tumor	LGG, RGNT	f79246
methylation class retinoblastoma	RETB	ff9755
methylation class schwannoma	SCHW	ffe1b2
methylation class melanotic schwannoma	SCHW, MEL	f7ca8a
methylation class craniopharyngioma, adamantinomatous	CPH, ADM	72d4c8
methylation class craniopharyngioma, papillary	CPH, PAP	43dad7
methylation class pituitary adenoma, ACTH	PITAD, ACTH	137d75
methylation class pituitary adenoma, FSH/LH	PITAD, FSHLH	44a199
methylation class pituitary adenoma, prolactin	PITAD, PRL	00b1a9
methylation class pituitary adenoma, STH sparsely granulated	PITAD, STHSPA	41ccbe
methylation class pituitary adenoma, TSH	PITAD, TSH	12d7c7
methylation class ependymoma, RELA fusion	EPN, RELA	d84c4b
methylation class chordoid glioma of the third ventricle	CHGL	b37dec
methylation class low grade glioma, subependymal giant cell astrocytoma	LGG, SEGA	ad6ef1
methylation class chordoma	CHORDM	fa8cff
methylation class Ewing sarcoma	EWS	ce69ec
methylation class hemangioblastoma	HMB	ed5cfc
methylation class melanoma	MELAN	0b4068
methylation class melanocytoma	MELCYT	102a46
methylation class lymphoma	LYMPHO	3c1b46
methylation class plasmacytoma	PLASMA	632f63
methylation class medulloblastoma, subclass group 3	MB, G3	0581be
methylation class medulloblastoma, subclass group 4	MB, G4	9dc3f5
methylation class medulloblastoma, subclass SHH A (children and adult)	MB, SHH CHLAD	2e80c6
methylation class medulloblastoma, subclass SHH B (infant)	MB, SHH INF	8bc0ff
methylation class atypical teratoid/rhabdoid tumor, subclass MYC	ATRT, MYC	006ead
methylation class atypical teratoid/rhabdoid tumor, subclass SHH	ATRT, SHH	009cea
methylation class atypical teratoid/rhabdoid tumor, subclass TYR	ATRT, TYR	44aeff
methylation class glioblastoma, IDH wildtype, H3.3 G34 mutant	GBM, G34	55c056
methylation class glioblastoma, IDH wildtype, subclass mesenchymal	GBM, MES	538533
methylation class glioblastoma, IDH wildtype, subclass midline	GBM, MID	27780d
methylation class glioblastoma, IDH wildtype, subclass MYCN	GBM, MYCN	48e948
methylation class glioblastoma, IDH wildtype, subclass RTK I	GBM, RTK I	68c62d

methylation class glioblastoma, IDH wildtype, subclass RTK II	GBM, RTK II	7af870
methylation class glioblastoma, IDH wildtype, subclass RTK III	GBM, RTK III	78e435
methylation class esthesioneuroblastoma, subclass A	ENB, A	aa4a1c
methylation class esthesioneuroblastoma, subclass B	ENB, B	ff8339
methylation class paraganglioma, spinal non-CIMP	PGG, nC	f5811d
methylation class pituitary adenoma, STH densely granulated, group A	PITAD, STHDNSA	01c3bd
methylation class pituitary adenoma, STH densely granulated, group B	PITAD, STHDNSB	14c5c9
methylation class low grade glioma, subclass posterior fossa pilocytic astrocytoma	LGG, PAPF	9d73c9
methylation class low grade glioma, subclass midline pilocytic astrocytoma	LGG, PAMID	b890e0
methylation class papillary tumor of the pineal region group A	PTPR, A	aede8a
methylation class papillary tumor of the pineal region group B	PTPR, B	bfff9c
methylation class pineoblastoma group B	PINT, PBB	b6dc95
methylation class ependymoma, posterior fossa group A	EPN, PFA	e30149
methylation class ependymoma, posterior fossa group B	EPN, PFB	df3854
methylation class ependymoma, spinal	EPN, SPINE	ff044c
methylation class ependymoma, YAP fusion	EPN, YAP	fe576c
methylation class CNS neuroblastoma with FOXR2 activation	CNS NB, FOXR2	518ccb
methylation class solitary fibrous tumor / hemangiopericytoma	SFT HMPC	ff6dfa
methylation class plexus tumor, subclass adult	PLEX, AD	7b3e0a
methylation class plexus tumor, subclass paediatric A	PLEX, PEDA	9d5723
methylation class plexus tumor, subclass paediatric B	PLEX, PEDB	754027
methylation class IDH glioma, subclass astrocytoma	AIDH	ecbf00
methylation class IDH glioma, subclass high grade astrocytoma	AIDH, HG	ddca11
methylation class IDH glioma, subclass 1p/19q codeleted oligodendroglioma	OIDH	ffe336
methylation class pineal parenchymal tumor	PINT, PPT	b4f79b
methylation class low grade glioma, ganglioglioma	LGG, GG	e4703a
methylation class pituicytoma / granular cell tumor / spindle cell oncocytoma	PITUI, SCO, GCT	58d8db
methylation class low grade glioma, subclass hemispheric pilocytic astrocytoma and ganglioglioma	LGG, PA/GGST	764e90
methylation class (anaplastic) pleomorphic xanthoastrocytoma	PXA	7c46c9
methylation class meningioma	MNG	d566d7
methylation class ependymoma, myxopapillary	EPN, MPE	b90f38
methylation class subependymoma, posterior fossa	SUBEPN, PF	dd052e
methylation class subependymoma, spinal	SUBEPN, SPINE	fa3b44
methylation class subependymoma, supratentorial	SUBEPN, ST	ff4156
methylation class pineoblastoma group A / intracranial retinoblastoma	PINT, PBA	cbf5af
methylation class low grade glioma, MYB/MYBL1	LGG, MYB	9159de
methylation class CNS high grade neuroepithelial tumor with BCOR alteration	HGNET, BCOR	739aca
methylation class anaplastic pilocytic astrocytoma	ANA PA	734ba9
methylation class CNS high grade neuroepithelial tumor with MN1 alteration	HGNET, MN1	ab94c1
methylation class infantile hemispheric glioma	IHG	d0bbd8
methylation class CNS Ewing sarcoma family tumor with CIC alteration	EFT, CIC	955c9d
methylation class control tissue, pituitary gland anterior lobe	CONTR, ADENOPIT	d3d3d3
methylation class control tissue, cerebellar hemisphere	CONTR, CEBM	bdbdbd
methylation class control tissue, hemispheric cortex	CONTR, HEMI	939393
methylation class control tissue, hypothalamus	CONTR, HYPTHAL	7e7e7e

methylation class control tissue, inflammatory tumor microenvironment *	CONTR, INFLAM	dbdbdb
methylation class control tissue, pineal gland	CONTR, PINEAL	696969
methylation class control tissue, pons	CONTR, PONS	545454
methylation class control tissue, reactive tumor microenvironment *	CONTR, REACT	d7d7d7
methylation class control tissue, white matter	CONTR, WM	a8a8a8

* Capper *et al*[2] dataset contains the following control tissue types: pituitary gland, anterior lobe, cerebellar hemisphere, hemispheric cortex, hypothalamus, pineal gland, pons, white matter, inflammatory tumour microenvironment and reactive tumour microenvironment. The Inflammatory tumor microenvironment and reactive tumor microenvironment were of particular interest to us as controls. Capper *et al*[2] write the following to describe these classes…

> "The methylation class 'control tissue, inflammatory tumor microenvironment' does not represent a distinct tumor class but rather a recurrently observed profile of mixed cell types with a high leukocyte fraction (often predominant granulocytic infiltrates). This is frequently observed in highly necrotic tumours, highly necrotic other tissues or when areas of extensive haemorrhage are sampled along with the tumor tissue of interest. Tumours with a pronounced granulocytic infiltrate due to other reasons can also get an elevated score for this class.'…
>
> "The methylation class 'control tissue, reactive tumor microenvironment' does not represent a distinct tumor class but rather a recurrently observed methylation profile of unclear status. A score for this class indicates that the extracted DNA is likely not suitable for classification by methylation profiling. The cases constituting this class are mostly low grade tumours (gangliogliomas or pilocytic astrocytomas) but also some high grade tumours. The cases share low tumor cell content and frequently show strong reactive changes (high proportion of reactive glial cell and frequently pronounced lymphocytic infiltration)."

Supplementary References

1. Newman, A. M. *et al.* Robust enumeration of cell subsets from tissue expression profiles. *Nat. Methods* **12,** 453–457 (2015).
2. Capper, D. *et al.* DNA methylation-based classification of central nervous system tumours. *Nature* **555,** 469–474 (2018).
3. Jeschke, J. *et al.* DNA methylation-based immune response signature improves patient diagnosis in multiple cancers. *J. Clin. Invest.* **127,** 3090–3102 (2017).
4. Franzen, J. *et al.* Senescence-associated DNA methylation is stochastically acquired in subpopulations of mesenchymal stem cells. *Aging Cell* **16,** 183–191 (2017).
5. Kozlenkov, A. *et al.* Differences in DNA methylation between human neuronal and glial cells are concentrated in enhancers and non-CpG sites. *Nucleic Acids Res.* **42,** 109–127 (2014).
6. Zhang, Y. *et al.* Genome-wide DNA methylation analysis identifies hypomethylated genes regulated by FOXP3 in human regulatory T cells. *Blood* **122,** 2823–2836 (2013).
7. Reinius, L. E. *et al.* Differential DNA methylation in purified human blood cells: implications for cell lineage and studies on disease susceptibility. *PLoS ONE* **7,** e41361 (2012).
8. Bakulski, K. M. *et al.* DNA methylation of cord blood cell types: Applications for mixed cell birth studies. *Epigenetics* **11,** 354–362 (2016).
9. Gervin, K. *et al.* Cell type specific DNA methylation in cord blood: A 450K-reference data set and cell count-based validation of estimated cell type composition. *Epigenetics* **11,** 690–698 (2016).
10. Mackay, A. *et al.* Molecular, Pathological, Radiological, and Immune Profiling of Non-brainstem Pediatric High-Grade Glioma from the HERBY Phase II Randomized Trial. *Cancer Cell* **33,** 829–842.e5 (2018).
11. Mackay, A. *et al.* Integrated Molecular Meta-Analysis of 1,000 Pediatric High-Grade and Diffuse Intrinsic Pontine Glioma. *Cancer Cell* **32,** 520–537.e5 (2017).
12. Northcott, P. A. *et al.* The whole-genome landscape of medulloblastoma subtypes. *Nature* **547,** 311–317 (2017).
