# Supplementary material for: Pediatric pan-central nervous system tumor analysis of immune-cell infiltration identifies correlates of antitumor immunity
Source: Nat Commun. 2020 Aug 28;11:4324. doi: 10.1038/s41467-020-18070-y (PMC7455736; doi:10.1038/s41467-020-18070-y)
Supplement: Supplementary file 2 — Descriptions of Additional Supplementary Files [file 41467_2020_18070_MOESM2_ESM.pdf]

Descriptions of additional supplementary files

Supplementary Data 1.

Description: Statistical results for tests of association between estimates of immune cell infiltration and survival or other clinico-pathological data. Data is presented as 4 sheets in an excel workbook each labelled according to test cohort.

Supplementary Data 2.

Description: Details of samples used to constitute test cohorts. Data is presented as 3 sheets in an excel workbook each labelled according to test cohort.
